# Photoacoustic imaging on its way toward clinical utility: a tutorial review focusing on practical application in medicine

**DOI:** 10.1117/1.JBO.28.12.121205

**Published:** 2023-06-08

**Authors:** Jonas J. M. Riksen, Anton V. Nikolaev, Gijs van Soest

**Affiliations:** Erasmus University Medical Center, Department of Cardiology, Rotterdam, The Netherlands

**Keywords:** photoacoustic imaging, clinical translation, quantitative imaging, systems engineering

## Abstract

**Significance:**

Photoacoustic imaging (PAI) enables the visualization of optical contrast with ultrasonic imaging. It is a field of intense research, with great promise for clinical application. Understanding the principles of PAI is important for engineering research and image interpretation.

**Aim:**

In this tutorial review, we lay out the imaging physics, instrumentation requirements, standardization, and some practical examples for (junior) researchers, who have an interest in developing PAI systems and applications for clinical translation or applying PAI in clinical research.

**Approach:**

We discuss PAI principles and implementation in a shared context, emphasizing technical solutions that are amenable to broad clinical deployment, considering factors such as robustness, mobility, and cost in addition to image quality and quantification.

**Results:**

Photoacoustics, capitalizing on endogenous contrast or administered contrast agents that are approved for human use, yields highly informative images in clinical settings, which can support diagnosis and interventions in the future.

**Conclusion:**

PAI offers unique image contrast that has been demonstrated in a broad set of clinical scenarios. The transition of PAI from a “nice-to-have” to a “need-to-have” modality will require dedicated clinical studies that evaluate therapeutic decision-making based on PAI and consideration of the actual value for patients and clinicians, compared with the associated cost.

## 1 Introduction

Medical imaging supports the diagnosis and treatment of a wide range of human diseases. The ability to visualize structures inside the human body is an invaluable means to establish the presence and exact location of disease processes and to assist in delivering the right treatment where it is needed. Photoacoustic imaging (PAI) is one such imaging modality. “Photoacoustics” has also been called “optoacoustics”; the terms are synonymous. It combines structural information with tissue composition by creating ultrasound images of absorbed optical energy. Thus, photoacoustic (PA) contrast derives from optical absorption, which can convey highly specific chemical information, in an image created from acoustic waves, which are subject to minimal tissue aberration.

As PAI relies on the detection of ultrasound, it shares many similarities with ultrasound echography, a modality with which it is frequently combined. Echography is a highly versatile and widely applied modality, indispensable in many diagnostic settings, ranging from obstetrics to vascular medicine and from orthopedics to cardiology. Specialized echographers are employed in radiology, cardiology, obstetrics, and other hospital departments for the acquisition, annotation, and reporting of the images.

Echo creates images from the reflections of ultrasound waves in the body. Reflections appear whenever a propagating acoustic wave encounters a change in acoustic impedance Z=ρ·c, the product of the density and the speed of sound that relate to bulk physical properties of tissue.^1^ Heterogeneity in those properties will generate contrast on echo. As a result, the modality is well suited for imaging morphology, such as fluid-filled cysts, the microarchitecture of tendons, the motion of the walls and valves of the heart, and the anatomy of the fetus. The different consistency of stiff lumps in for instance the breast or liver may be visible on ultrasound as well. Without specifically targeted contrast agents, the molecular composition of tissues is not accessible to echo imaging.^2^ Tissue type identification in echo relies critically on the interpretation of image brightness patterns, which requires extensive expertise in the recognition of artifacts and variable appearance of anatomical structures depending on the imaging geometry. PAI adds a completely different form of contrast, based on tissue optical properties, which is not accessible to purely ultrasonic imaging.

PAI creates images from ultrasound that is generated inside the tissue. The transient thermo-elastic expansion that follows the absorption of a short pulse of optical energy creates a distributed acoustic source in the illuminated volume. PAI attempts to reconstruct the spatial structure of that source, by the detection of the ultrasound signals in time. To this end, PAI may take many cues from echography, in instrumentation as well as signal processing and image reconstruction. It does, of course, require a light source, in addition to an ultrasound system, but if this can be added to an imaging system unobtrusively and affordably, there is wide-ranging potential for PAI to add color to familiar grayscale structural echo images, the color encoding molecular or chemical tissue composition. This is the angle we pursue in this tutorial review: PAI as “echo with color,” aiming for broad applicability, affordability, and maximum potential for acceptance by echographers and different communities of physicians familiar with ultrasonic imaging.

The ability to gather optical contrast from inside the human body at depths significantly greater than the optical scattering mean free path makes PAI a highly attractive imaging technology that has been the focus of an intense research effort over the past two decades. It enables monitoring of hemoglobin content^3^ and oxygen saturation in blood^4^^,^^5^ and detailed biochemical characterization by means of spectroscopy of tissue components.^6^^–^^9^ Non-absorbing structures may be visualized with the aid of exogenous contrast agents, such as molecular dyes showing the lymphatic system^10^^,^^11^ and by antibody-targeted absorbing particles or molecules that can identify the location of specific receptors or molecules.^12^ Inflammation has been visualized both by increased vascularity^13^ and with the aid of antibody constructs.^14^ These are just a few examples of the myriad phenomena to which PAI has been applied.

A substantial body of PAI literature exists, which is predominantly technical or preclinical in nature. A Pubmed search on the terms “photoacoustic* OR optoacoustic*” yields 9392 records (search performed on March 7, 2023), of which a striking number of 836 are reviews, and quite few are clinical studies. These numbers illustrate the challenge that the field is facing: PAI has been an “emerging,” or “promising,” technology for more than two decades. Many potential applications of PAI have been explored, and it has succeeded in visualizing aspects of human diseases and disease models that are not readily accessible by other means. Since 2015, the conference “Photons Plus Ultrasound” is the largest meeting at Photonics West, the flagship symposium of the international society for optics and photonics (SPIE).

On the other hand, PAI remains a relatively complex technology to translate to clinical use. We will see that the imaging physics of PAI puts stringent requirements on the ultrasound hardware and specifications of the light source, in order for the acquired data to be robust and of high quality. Interpretation and quantification of the data require a thorough understanding of tissue optics and artifacts arising in image formation. For the technology to mature and evolve from a nice-to-have to a need-to-have in a number of well-defined clinical scenarios, not only will the images have to be informative and clear, but also the accessibility and ease of use of imaging systems will be at least as important.

These considerations are what we took as a starting point for this tutorial review, and what sets it apart from the existing literature: we aim here to make a connection to (wanted and unwanted) image features to the imaging physics. Today, a thorough understanding of the physical principles that affect image formation remains important for correct image interpretation. The achievements of clinical PA imaging can be found in many reviews,^15^^,^^16^ but the background and practical steps required to actually distill valuable images out of the acquired data has not received as much attention. We outline these steps and show how they are being applied in selected clinical and translational scenarios.

The history of PAI has been reviewed in depth recently.^17^ Since the publication of that review, the field has progressed markedly, in technical prowess but also in its approach toward clinical applications. Most significantly, two commercial systems for clinical imaging have been released that received approval from the United States Food and Drug Administration (FDA) and CE-mark in Europe. Of the many scientific advances toward clinical application that we will not discuss in more detail here, imaging of the human brain with contrast, field-of-view, and resolution similar to magnetic resonance imaging (MRI) in people with a hemicraniectomy stands out as a remarkable technical achievement.^18^ Research on light sources^19^^,^^20^ and ultrasound detectors^21^^,^^22^ aims to improve the sensitivity, portability, cost, and size of PAI systems, which are all incredibly important for the eventual role of PAI in clinical practice.

This tutorial review is structured as follows: in Sec. 2, we review the physical principles of PAI. These have been presented in many places.^23^^–^^25^ Nevertheless, for comprehensiveness, consistency in symbols and terminology, and for emphasizing the salient consequences for practical, clinical imaging, it is useful to include the material. We touch upon tissue optics, acoustic propagation, and image reconstruction methods. We also explain common artifacts and their impact on image interpretation and quantification at some length, as understanding these is important for accurate image interpretation. Then, in Sec. 3, we address the technical implementation of PAI, building upon the imaging physics discussed before. The practical implications of optimal choices of, for instance, wavelength, receive frequency and bandwidth, and pulse duration will be laid out. We emphasize solutions that may be more conducive to clinical translation by their versatility, accessibility, mobility, and robustness. Section 4 considers safety and standards under development. Subsequently, Sec. 5 discusses a few examples of clinical and translational research with PAI, specifically focusing on studies that leverage the well-established acquisition methods in ultrasound echography. We conclude the review by discussing an outlook on PAI in future clinical diagnostic settings.

## 2 General Principle of Photoacoustic Imaging

**Fig. 1:**
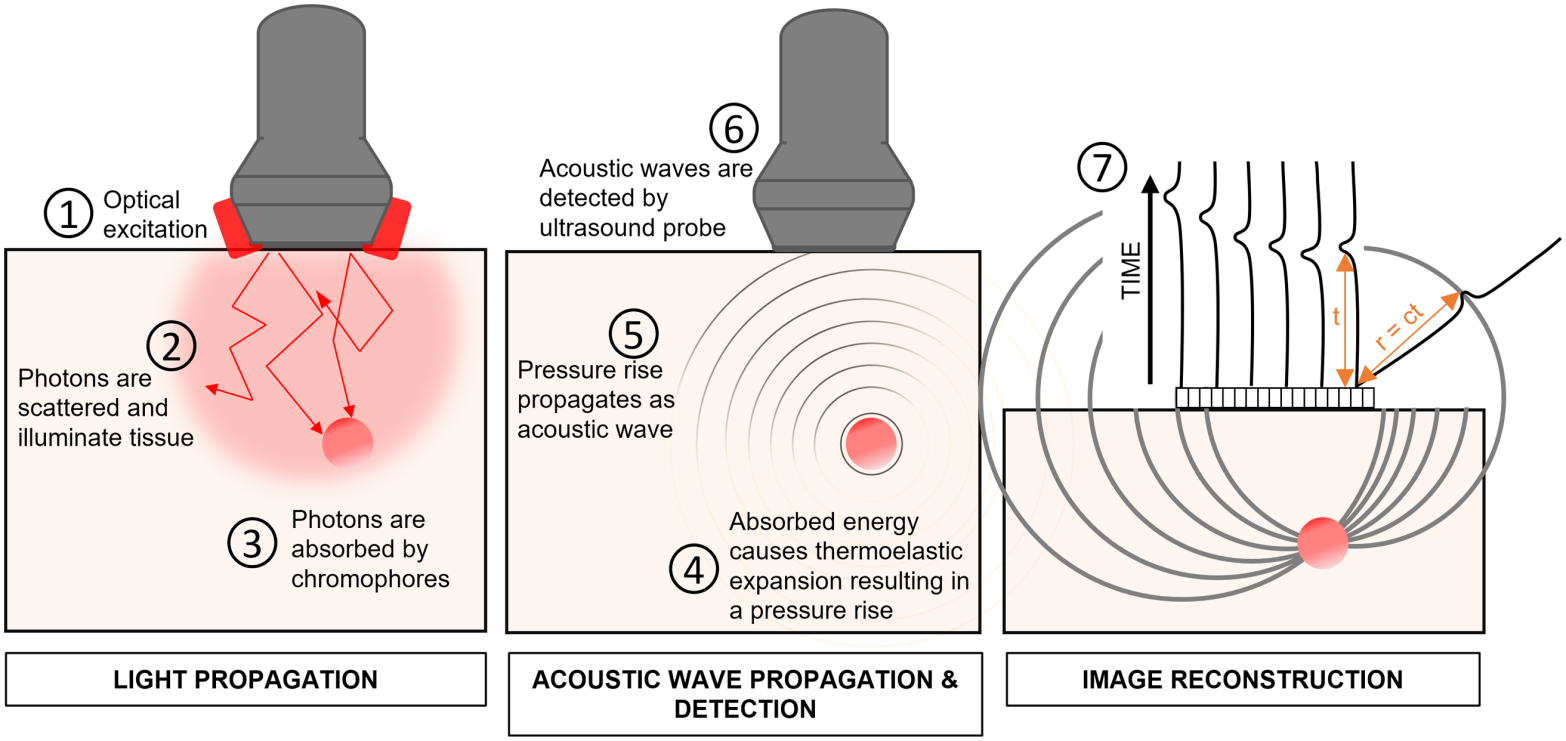
Principle of PAI. (1) Light emitted from a source illuminating the volume of interest propagates through the tissue, which is generally turbid. (2) Scattering distributes the light through the tissue but also attenuates it as it reaches greater depths. (3) Chromophores absorb light. (4) The absorbed optical energy thermalizes and causes a transient elastic response that generates a local pressure rise. (5) The pressure change sets off an acoustic wave that propagates through the tissue, and can be (6) detected by an ultrasound probe. If an ultrasound array is used, an image of the absorbed optical energy can be reconstructed (7) by dedicated algorithms, analyzing the acoustic delays of the signals received by the individual array elements. Modified from Ref. 26 with permission of the author.

PAI is a form of acoustic imaging. The image is created from signals received by an ultrasound transducer, which converts an impingent acoustic pressure to a current with a certain sensitivity and frequency response. The pressure at the transducer arrives there by propagation from an acoustic source in the medium, according to the wave equation. The source in question is created by thermalization of optical energy, absorbed by chromophores inside the medium, which are our prime object of interest in PAI. The process is shown in Fig. 1.

The signal generation in PAI thus can be separated into an optical forward problem (describing the spatial distribution of the delivered light), thermalization of the absorbed energy, and an acoustic forward problem (describing how the pressure relaxation propagates to the transducer). An image can be created by processing the acoustic delays of the acquired signals, where the distance r from source to detector is r=ct, the product of the speed of sound and the propagation delay.

A full quantitative reconstruction requires solving the optical and acoustic inverse problems, which are generally ill-posed, and in the case of the optical part, nonlinear and even non-local. In this section, we present the mechanisms involved in signal generation and discuss their implications for image reconstruction. In the following sections, we broadly follow the treatment as introduced in Refs. 27–29.

### 2.1 Heat-to-Pressure Conversion

A thermo-acoustic signal is generated when heat H is deposited in an elastic medium with density ρ and specific heat at constant volume $C_{v}$, leading to a temperature rise

$$\Delta T=\frac{H}{\rho C_{v}}.$$ (1)

The thermal deposition varies in space x and time t, so H=H(x,t), an energy density with units $J\;m^{-3}$. If the deposition of heat happens sufficiently rapidly, heat transport can be ignored during heating and the temperature rise is adiabatic. This condition of thermal confinement is satisfied if the heating time is shorter than $t_{\mathrm{th}}=\frac{d^{2}}{4\chi }$, for a heated region size d and thermal diffusivity χ.^27^

The change in material density that follows upon heat deposition is the difference between compression caused by a rise in pressure and rarefaction caused by thermal expansion:

$$\Delta \rho =\rho \kappa_{T}\Delta p-\beta \rho \Delta T,$$ (2)

$\kappa_{T}$ denoting the compressibility of the material and β is the thermal expansion coefficient. Introducing another time scale, the acoustic transit time of the heated region $t_{\mathrm{ac}}=\frac{d}{c}$. If the heat deposition happens faster than this time scale, there is no density change during the heating process; Δρ=0. If this condition, which is called stress confinement, is satisfied, the pressure has no time to relax during the heat deposition and can be treated as instantaneous and the space and time dependences of the heat deposition function can be separated:

H(x,t)=H(x)δ(t). (3)

At the length scales investigated with biomedical imaging, stress confinement is usually more restrictive than thermal confinement: the thermal transport time over a heated object of 100  μm is around 16 ms in biological media, whereas the acoustic transit time is ∼65  ns.

The powerful assumption of stress confinement greatly simplifies the influence of the thermalization of the deposited energy on the pressure field that is generated. Under stress confinement, we can compute the resulting pressure rise, which sets off the acoustic wave by which we will try to reconstruct the heating distribution, from setting Δρ=0 in Eq. (2), inserting Eq. (1), and using the thermodynamic identity $\kappa_{T}=\frac{C_{p}/C_{v}}{\rho c^{2}}$, with $C_{p}$ denoting the specific heat at constant pressure (expressed in Pa):

$$\Delta p(x)=\frac{\beta c^{2}}{C_{p}}H(x).$$ (4)

For later reference, we introduce the so-called Grüneisen parameter

$$\Gamma =\frac{\beta c^{2}}{C_{p}},$$ (5)

a dimensionless number that describes the efficiency of heat-to-pressure conversion. With this substitution, the initial pressure rise $p_{0}(x)$, happening at t=0, may be written as

$$p_{0}(x)=\Gamma H(x).$$ (6)

Note that the heterogeneity of thermal and mechanical parameters of different tissue components generally leads to a nonuniform Grüneisen parameter Γ(x), which modulates the pressure field independent of the deposited heat. The heterogeneity of Γ is commonly ignored, although variation between different biological materials can be significant: $\Gamma_{\mathrm{water}}=0.12$ while $\Gamma_{\mathrm{fat}}\approx 0.8$ at room temperature:^30^ the large heat capacity of water leads to lower pressure generation. Because of the temperature dependence of β and c (and to a lesser degree, of $C_{p}$), Γ is generally temperature dependent as well. For quantitative PAI in heterogeneous media, knowledge of the Grüneisen parameter is essential.

While in a large majority of scenarios (nanosecond pulses, <10  MHz detection frequencies) stress confinement is an excellent assumption, it is important to realize that the duration of the excitation pulse $t_{p}$ determines the smallest length scale $d_{\min}=ct_{p}$ where stress confinement still holds, and the largest frequency $f_{\max}$ that can be generated. This can be understood as follows: during adiabatic heating of a point absorber, the instantaneous thermal expansion [the second term in Eq. (2)] follows the heating function, which is the temporal profile of the applied pulse, generating the compressive half of the pressure wave. After the pulse, the elastic medium rebounds, leading to rarefaction in the trailing half of the wave. Thus, the pressure wave principally has a period$\;2t_{p}$ and a maximum frequency $f_{\max}=\frac{1}{2t_{p}}$. This effect is visible in experiments with relatively longer pulses, small targets, and high detection frequencies.^31^

### 2.2 Optical Forward Problem

Energy is delivered to a medium under study in the form of light, introducing an optical fluence F (measured in $J\;m^{-2}$) throughout the illuminated volume. Absorption by chromophores with an absorption coefficient $\mu_{a}$ (in $m^{-1}$) leads to a heat deposition

$$H(x,t)=\mu_{a}(x)F(t),$$ (7)

under the assumption that all absorbed energy is converted into heat. This is true for most biological media but not for many popular contrast agents that have been developed for their fluorescent properties. Fluorescent dyes and nanoparticles emit part of the absorbed energy at a longer wavelength, which may or may not be subsequently absorbed by a tissue component. Also, this re-emission may take place over timescales from nanoseconds ($\ll t_{\mathrm{ac}}$) to microseconds ($>t_{\mathrm{ac}}$, usually). For the present discussion, we ignore this effect but it is worthwhile to observe this subtlety when dealing with exogenous contrast agents.

Optical absorption by electronic or vibrational molecular transitions happens on very short time scales compared to even $t_{\mathrm{ac}}$ and thus can be treated as instantaneous. If the illuminating field is pulsed with a duration

$$t_{p}\ll t_{\mathrm{ac}},$$ (8)

stress confinement holds so F(t)=Fδ(t), and we can write

$$p_{0}(x)=\Gamma \mu_{a}(x)F,$$ (9)

which describes the relation between the PA source pressure and optical absorption. Given a homogeneous fluence, which may be a reasonable assumption for weakly scattering media, and small absorbing targets relative to the reduced scattering length $\ell_{s}^{\prime }$, $\mu_{a}(x)$ is proportional to $p_{0}$. Thus, the absorption coefficient can be reconstructed straightforwardly from the pressure that has propagated to the detector.

**Fig. 2:**
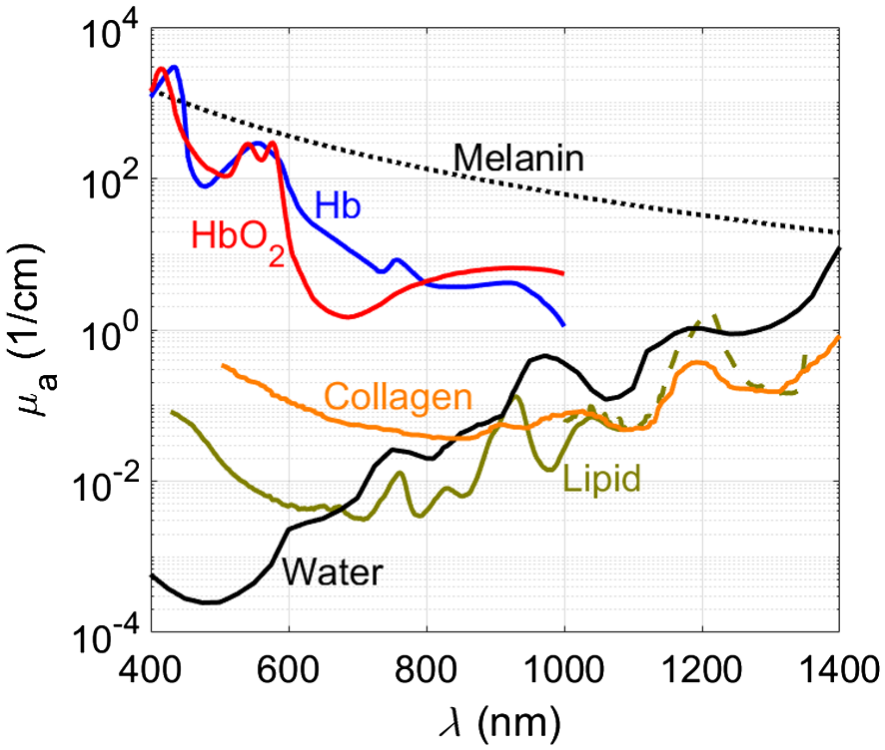
PA spectra in terms of absorption coefficient of endogenous chromophores: melanin,^32^ oxygenated (${\mathrm{HbO}}_{2}$) and non-oxygenated (Hb) hemoglobin at the concentration of 150 g/l,^33^ collagen,^34^ lipid (the regions depicted with a solid and a dashed line are from Refs. 35 and 36 respectively), and water. All spectra except collagen and lipid (dashed line) are available from Ref. 37.

Often, multiple different optical absorbers, or so-called chromophores, are present. These can often be separated using their known and unique absorption spectra (see Fig. 2) by illuminating the tissue with multiple wavelengths:

$$p_{0}(x,\lambda )=\Gamma \mu_{a}(x,\lambda )F.$$ (10)

A family of techniques known as “spectral unmixing,” which is discussed in a separate section, aims to separate the different absorption spectra in a series of PA acquisitions.

**Fig. 3:**
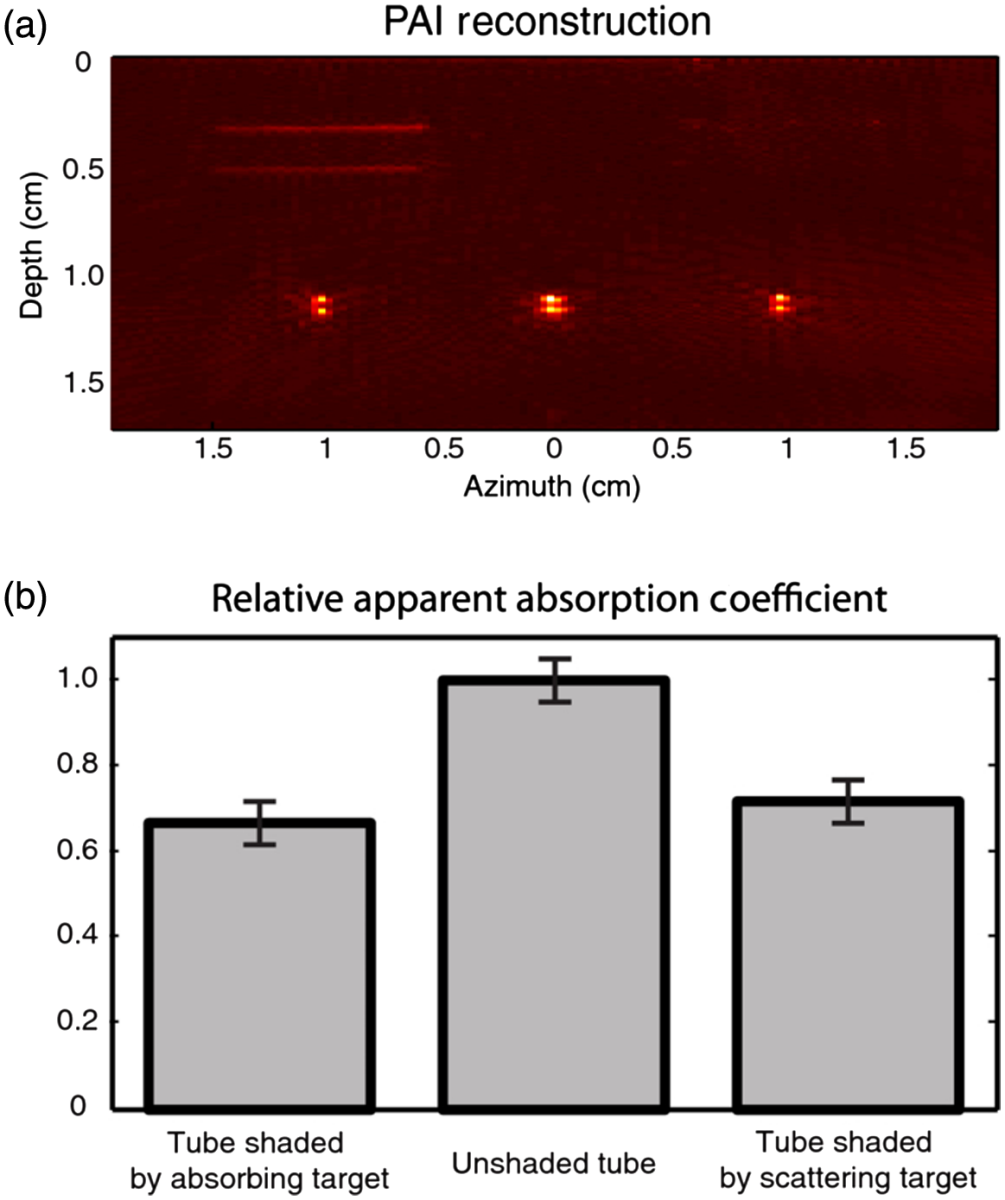
(a) Image of three identical India ink filled capillary tubes, illuminated from above. The tubes are shielded by an absorbing layer (left), which is visible as two boundaries in the image; unshielded (middle); and shielded by a scattering layer (right), which is invisible in the image. (b) The apparent absorption coefficient varies as a result of changes in fluence; note that scattering and absorption have similar attenuating effects. Reproduced from Ref. 38 with permission.

In practice, obviously, many biological tissues are not optically transparent and the combined effects of scattering and absorption lead to a non-uniform fluence distribution F(x). The optical attenuation coefficient is the sum of the scattering and absorption coefficients; $\mu_{t}=\mu_{s}+\mu_{a}$. Optical attenuation is the most important limitation in PAI since it constrains the viewing depth and breaks the direct relation between spatial variations in $p_{0}$ and $\mu_{a}$. A variable amount of optical attenuation in the path from the light delivery device to the imaging target affects the initial pressure generated by that target, as illustrated in an instructive experiment shown in Fig. 3.^38^ Both the scattering $\mu_{s}(x^{\prime })$ and absorption $\mu_{a}(x^{\prime })$ can similarly reduce the fluence at a deeper location x, but only absorption produces a PA signal, as visible by the lines at the top left of Fig. 3(a). $H(x)=\mu_{a}(x)F(x;\mu_{a}(x^{\prime }),\mu_{s}(x^{\prime }))$ becomes a nonlinear and non-local function of the absorption distribution.^29^ The measurement of the PA signal thus does not allow a unique reconstruction of the tissue optics that affect the image. To make matters worse, the fluence distribution is generally not only spatially but also wavelength-dependent since both components of attenuation, absorption and scattering, vary with optical wavelength in biological media.

Now, if we want to quantitatively recover the absorption properties of any imaging targets, the fluence distribution must be known. Depending on the accuracy and detail level needed, and on the scattering strength and heterogeneity of the tissue, many approaches have been proposed to estimate the fluence distribution.

The simplest estimates of fluence distribution are based on homogeneous scattering and absorption properties of the tissue. In weakly scattering media, a first guess of the fluence as a function of depth z can be obtained by applying Beer’s law, $F(z)=F_{\mathrm{in}}e^{-\mu_{t}z}$. For biological media, scattering dominates absorption for wavelengths between 400 and 1400 nm.^39^ Then, the transport mean free path (or effective scattering length) is the distance by which the directionality of an impingent light beam is lost: $\ell_{s}^{\prime }=\frac{\ell_{s}}{1-\cos\;\theta }$, where $\ell_{s}=\mu_{s}^{-1}$ and cos θ is the average cosine of the scattering angle. Beyond this distance, the fluence distribution can be considered diffuse, and the effective penetration depth can be estimated as $L_{\mathrm{eff}}=[3\mu_{a}(\mu_{a}+\mu_{s}^{\prime }){]}^{-\frac{1}{2}}$.^40^

Highly refined methods for fluence estimation have been developed, and their utility for PAI has been comprehensively reviewed.^29^ Broadly, different approaches to solving the radiative transfer equation exist, varying from diffusion models that deal best with homogeneous media^41^ to stochastic Monte Carlo models^42^^–^^44^ that allow highly detailed (but poorly generalizable) representations of heterogeneous optical properties in biological systems. Forward optical models can predict the fluence, given a set of tissue optical parameters. If those parameters are unknown, inversion methods must be applied, which can be based on the measurement of the diffusely scattered light.^38^

### 2.3 Acoustic Wave Propagation

In any PAI scenario, the spatially distributed source pressure $p_{0}$ must be reconstructed from the received pressure time trace at the location of the detector. We now turn to the problem of acoustic propagation from source to detector. In the case of stress confinement, the light pulse is transformed instantaneously into the spatial pressure variation $p_{0}$ at position$x^{\prime }$. The pressure variation propagates as a sound wave according to the wave equation:

$$(\nabla^{2}-\frac{1}{c^{2}}\frac{\partial^{2}}{\partial t^{2}})p(x,t)=-\frac{1}{c^{2}}p_{0}(x^{'})\frac{\partial \delta (t)}{\partial t}.$$ (11)

In this tutorial, we assume the wave is propagating in a homogeneous fluid, which implies only the presence of longitudinal waves; transverse waves are not supported in a fluid medium. The equation can be solved using a Green’s function $G(x,t;x^{\prime },t^{\prime })$ describing the wave propagation from $x^{\prime }$ at $t^{\prime }$ to x at

$$(x,t;x^{\prime },t^{\prime })=\frac{\delta (|x-x^{\prime }|-c(t-t^{\prime }))}{4\pi |x-x^{\prime }|},$$ (12)

$$p(x,t)=\frac{1}{c^{2}}\overset{\infty }{\underset{0}{\int }}\int_{V}p_{0}(x^{\prime })G(x,t;x^{\prime },t^{\prime })\;\delta^{\prime }(t^{\prime })dx^{\prime }dt^{\prime }.$$ (13)

Taking into consideration $\int_{0}^{\infty }f(t)\delta^{\prime }(t-t_{0})dt=-f^{\prime }(t_{0})$, and in Eq. (12) $\partial G/\partial t^{\prime }=-\partial G/\partial t$, Eq. (13) can be simplified to

$$p(x,t)=\frac{1}{c^{2}}\int_{V}p_{0}\frac{\partial G}{\partial t}(x,t;x^{\prime },t^{\prime })dx^{\prime }.$$ (14)

**Fig. 4:**
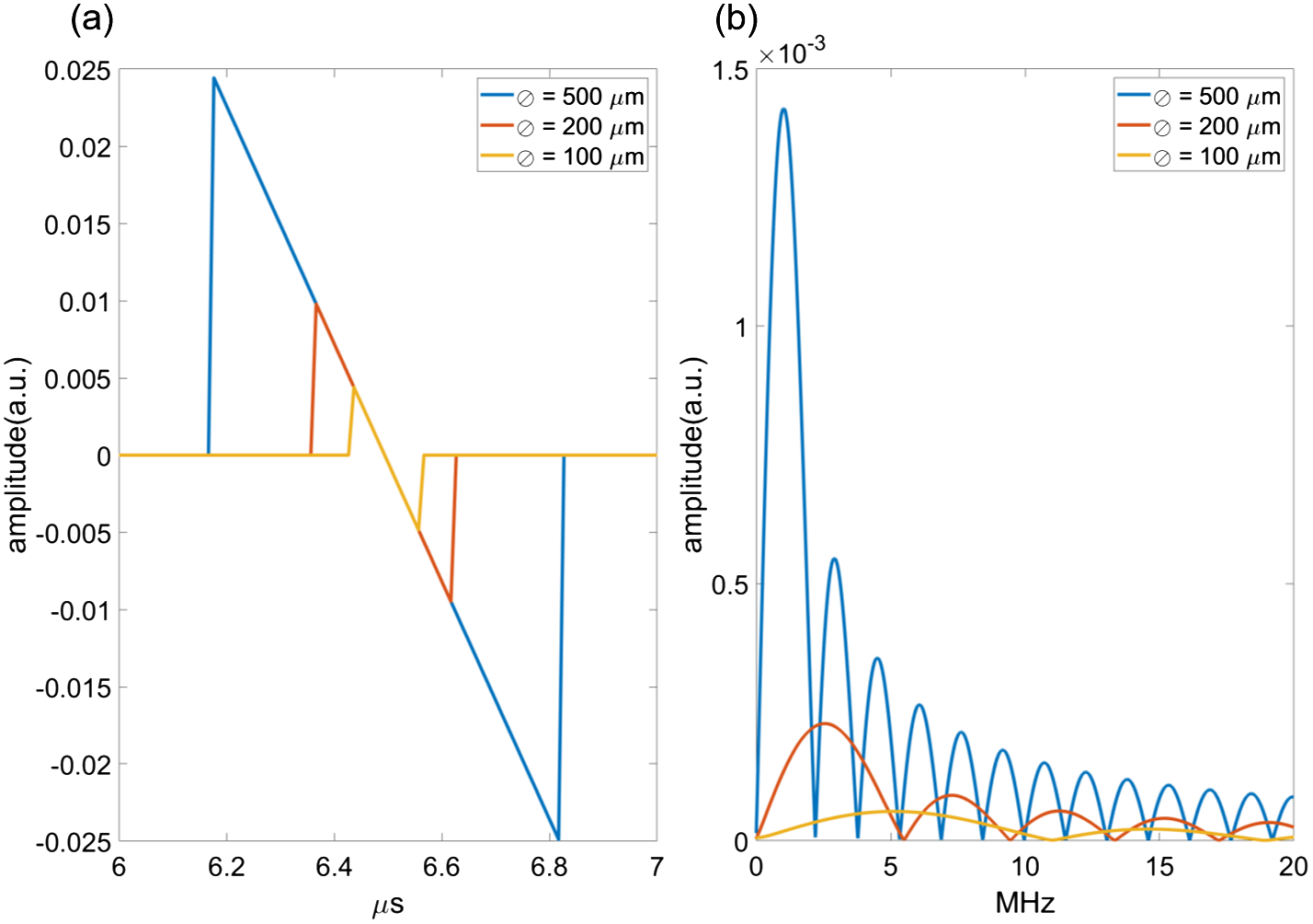
(a) Time- and (b) frequency domain solutions for Eq. (14) for spherical absorbers with diameters of 500, 200, and 100  μm.

Equation (14) can be solved analytically for simple geometries such as a slab, sphere, or cylinder.^45^ For more complex geometries, the equation can be solved numerically with tools such as k-wave.^46^ The solution for spherical PA absorbers of different diameters is shown in Fig. 4, showing the famous “N-wave,” where the length of the wave corresponds to the diameter of the sphere. The largest absorber emits an acoustic wave with the longest wavelength, and thus lower frequencies and narrower bandwidth than the smaller absorbers, as shown in Fig. 4(b). At the same time, the largest source emits a PA signal with the largest power. In practice, realistic samples comprise absorbers of various sizes. Consequently, such samples emit PA signals of a wide frequency range where the signals emitted by small and large absorbers belong to the high and low frequency ranges, and where low frequency signals tend to contain the largest power.

Inversion of Eq. (14) to yield $\mu_{a}$(using Eq. (10)) given the recorded pressure p is the essential image formation step in PAI. The image information is carried by the acoustic wave, which retains (most of) its spatial coherence properties and thus allows the application of amplitude and phase (or equivalently, path length) for image formation. The optical field coherence is scrambled by (multiple) scattering and thus cannot provide a detailed image, but that is not required: only the absorbed optical power matters to initiate PA wave generation.

Equation (14) has been derived under the assumption of stress confinement, treating the excitation as a delta function in time. The effect of finite pulse duration can be accounted for by convolving the resulting waveform p(x,t) with the time dependence of the heating function. This also introduces the frequency cutoff discussed at the end of Sec. 2.1.^28^

### 2.4 Acoustic Forward Problem

In a realistic situation, the acoustic wave propagates through an attenuating inhomogeneous medium, where acoustic properties, such as speed of sound and density, are variable. Besides, the wave is detected by an ultrasound transducer of limited bandwidth. Such factors have an impact on the reconstruction of the pressure distribution $p_{0}$ from the recorded pressure  p.

While propagating, the acoustic wave is subject to frequency-dependent acoustic attenuation, usually modeled as $p(z_{2})=p(z_{1})e^{-\alpha f^{y}(z_{2}-z_{1})}$. Here, f is the wave frequency. The attenuation coefficients α (measured in $\mathrm{dB}/{\mathrm{MHz}}^{y}\cdot \mathrm{cm}$) and y are the properties of the medium in which the sound wave propagates.^47^ The acoustic attenuation increases with frequency and, unlike in optics, is dominated by absorption. Acoustic attenuation can limit the PAI depth, similar to optical attenuation, but it does so in a frequency-dependent manner. Compared with large targets, the visibility of small features decreases faster with depth in an acoustically attenuating medium. The effects of acoustic wave attenuation can be mitigated partially by increasing the sensitivity of the ultrasound transducer and its front-end circuit by a technique called time-gain compensation (TGC).^48^

Finally, the propagation of the acoustic wave is impacted by a varying speed of sound within the sample. Image formation algorithms frequently assume a fixed value for the speed of sound c (normally at 1540  m/s). The mismatch between the assumed and the real c results in aberration, i.e., errors in arrival-to-the-sensor-time points. The errors lead to wrongly estimated time delays (see Fig. 1) required for image formation and can substantially deteriorate the image quality. Such errors are well-known from echo imaging, for instance, severe aberration occurs in ultrasonic imaging of the heart ($c_{\mathrm{cardiac}}=1560\;m/s$) with a thin layer of subcutaneous fat ($c_{\mathrm{fat}}=1440\;m/s$) present.^49^ Acoustic aberration is one of the most challenging problems in both echography and PAI.

Techniques to reconstruct the speed of sound using either PA signals^50^^,^^51^ or ultrasound tomography^52^ have been applied successfully to create sharp images in heterogeneous acoustic media. Computational approaches have borrowed from autofocus algorithms,^53^ performed iterative sound speed reconstructions,^54^ or applied deep learning methods.^55^

### 2.5 Photoacoustic Image Formation

PA image formation is an inverse problem where the recorded pressure traces p at the transducer are combined to estimate the initial pressure distribution $p_{0}$. For single-element transducers, which may have a role in scanning microscopy or endoscopy, the propagation delay t is usually directly converted into the propagation distance according to r=ct. Knowledge of the beam profile, which is the same as the distribution of receive sensitivity in space, may be applied to deconvolve the image created by stacking adjacent lines, creating sharper images.^56^^,^^57^

The image quality is conventionally measured in terms of resolution, signal-to-noise ratio (SNR), and contrast-to-noise ratio (CNR).^58^ The quality metrics depend dramatically on the selection of the beamforming algorithm.^59^ Choosing a suitable algorithm is always a trade-off between the computational efficiency and the image quality and should be determined by the application. Next, the image quality depends on the speed of sound homogeneity (see Sec. 2.4) and laser pulse duration (see Sec. 2.1). Finally, the resolution is determined by the transducer sensitivity, number of elements, and configuration.

#### 2.5.1 Beamforming techniques

In photoacoustic tomography (PAT), pressure recordings from different elements in an array are combined to form an image. Several popular methods exist to achieve this, such as the commonly used filtered back-projection (FBP) and delay-and-sum (DAS)^60^ algorithms.

**Fig. 5:**
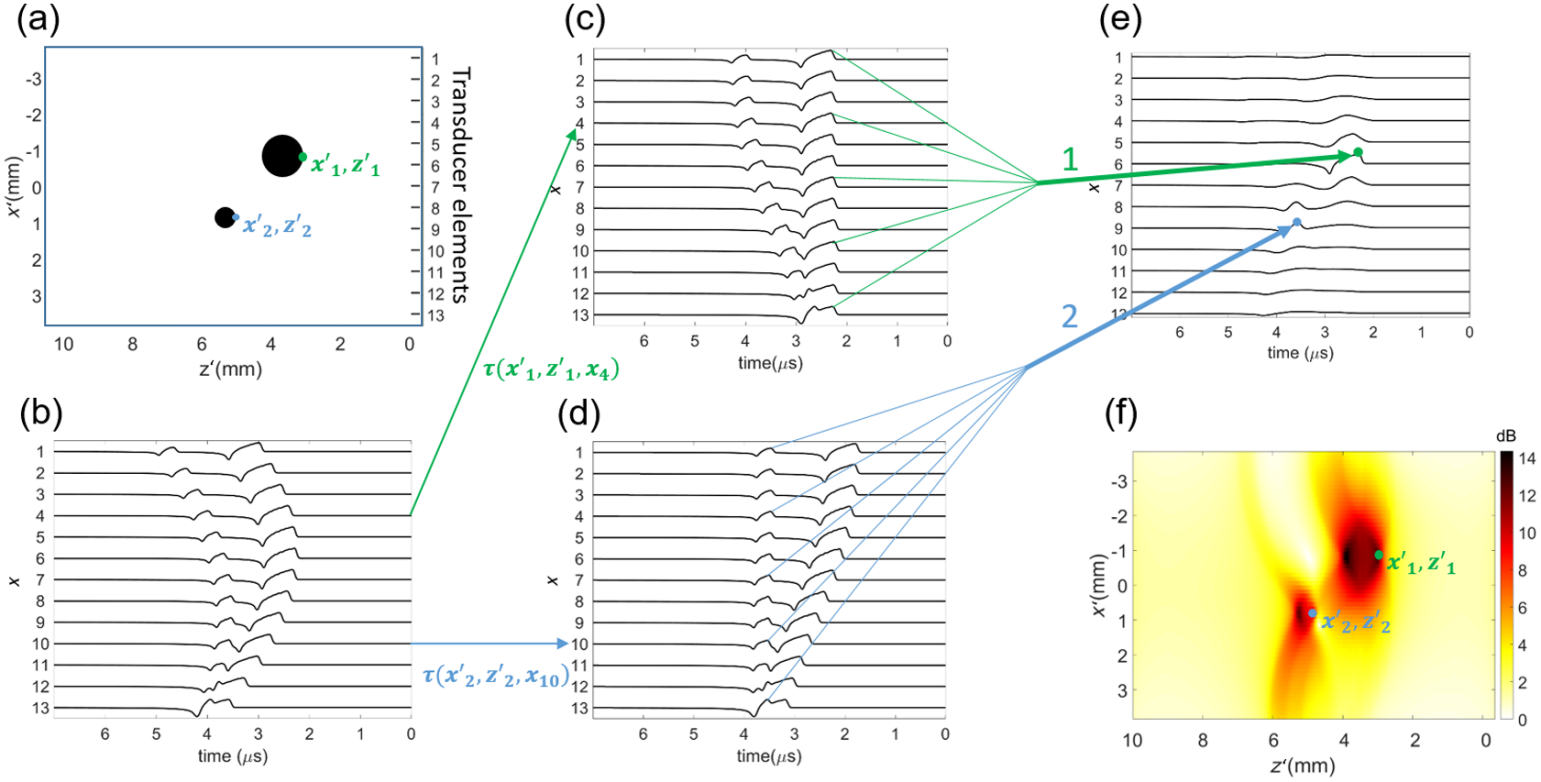
Principle of DAS algorithm. (a) The initial pressure distribution p0. (b) The pressure traces p received by the ultrasound transducer elements located at x. (c), (d) To reconstruct the initial pressure distribution at points $x^{\prime }1,z^{\prime }1$ depicted with green and $x^{\prime }2,z^{\prime }2$ depicted with blue, a different set of delays τ is to be applied. (e) The delayed traces at each channel are added together to form the beamformed signal. (f) Finally, the beamformed signal is enveloped and log compressed for displaying.

The principle of DAS for linear ultrasound transducer arrays is shown in Fig. 5. The initial pressure $p_{0}$ emitted at location $\left(x^{\prime },z^{\prime }\right)$ will arrive at transducer element I located at $\left(x_{i},0\right)$ with a time delay

$$(\tau^{\prime },z^{\prime },x_{i})=\frac{\sqrt{z^{\prime 2}+(x^{\prime }-x_{i})}-z^{\prime }}{c},$$ (15)

specifying a set of element-specific delays for each pixel in the reconstructed volume. To restore $p_{0}$ at ($x^{\prime },z^{\prime }$), DAS compensates for the delays and sums the pressures$\;p_{i}$ recorded by each element:

$$p_{0}(x^{\prime },z^{\prime })=\sum_{i\leq N}p(x^{\prime },\frac{z^{\prime }}{c}-\tau (x^{\prime },z^{\prime },x_{i})),$$ (16)

where N is the number of transducer elements and $x^{\prime }\in x$. DAS is widely used for beamforming conventional echo-mode ultrasound data with the only difference being that the distance in PA is proportional to one-way time-of-flight. Consequently, the majority of beamforming algorithms for echo-mode can also be applied for PAI. Delay-multiply-and-sum (DMAS) is a modification of DAS where all possible pair products of delayed pressure traces are summed to form an RF-line.^61^ DMAS and its modifications^62^^,^^63^ deliver images of greater contrast, which comes at the cost of non-linearity.

The filtered, or universal, back-projection method makes use of the initial spherical emission $p_{0}$, as projected on the ultrasound transducer array.^64^^,^^65^ The received pressure per element is high-pass filtered with a ramp filter in the frequency domain and then projected back to the source. The filter amplifies high frequencies to enhance the reconstruction of edges and small features. Originally developed for x-ray computed tomography, FBP is frequently applied in tomographic imaging with a large (solid) angle coverage of the receiving transducer.^64^^,^^66^ Image fidelity is impaired when applied to linear array data.^67^

Another popular method is an algorithm called “time reversal,” which simulates a source at the detector location and “plays back” the recorded pressure.^68^ This method can deliver very high-quality images but is computationally inefficient and which limits its utility for real-time imaging applications. Model-based methods formulate image formation as an optimization problem in which the chromophore distribution is optimized until its simulated PA response matches the recorded pressure p.^69^ Model-based methods can be applied to regularize image reconstruction in limited-view geometries, producing high-resolution images with a reduced artifact level from incomplete PA data.^70^^–^^72^

Adaptive beamforming methods substantially outperform conventional beamforming methods in terms of resolution and CNR by locally minimizing the noise in the image.^73^ For instance, the full-wave reconstruction iteratively optimizes the initial pressure distribution $p_{0}$.^74^ At each iteration, the forward wave propagation is simulated. The method aims to minimize the difference between real and simulated recorded pressure traces p. The image formation may use only part of the transducer elements, emphasizing sparsity by regularization with the $L_{1}$ norm of the solution. This method enables imaging of larger areas using fewer transducer elements. It must be noted that, like DMAS, adaptive beamforming, minimum variance beamforming,^73^ and coherence-based methods^75^^,^^76^ may be nonlinear and thus do not return a quantitative estimate of $\mu_{a}$.

#### 2.5.2 Image display and image quality

Before displaying the beamformed data, it has to be processed into a final image. Several choices in the post-processing algorithms determine the appearance, dynamic range, CNR, and feature visibility.

The beamformed data retain the oscillatory wave signal that is present in the received pressure signal. This is often called radiofrequency (RF) data, and images are sometimes analyzed in this form. For a more readily interpretable display, the beamformed RF data can be converted to envelope data by applying, for instance, the Hilbert transform and taking the absolute value. This nonnegative waveform can then be log-compressed to a dB scale, although PA data are frequently displayed in a linear scale. By adding transparency to the low-amplitude areas of PA images, they can be directly overlaid on the interleaved ultrasound images acquired in the same geometry.

PA signals can be relatively weak and thus noisy. Image formation algorithms as discussed in Sec. 2.5.1 are inherently noise-suppressing since they combine (parallel) measurements from different acquisition channels to reconstruct one pixel. Bandpass filtering is a straightforward approach to removing electronic noise outside the transducer frequency band.

If PA signals remain too weak relative to the system noise even after frequency filtering, or noise is actually due to environmental signals picked up by the (band-limited) receive electronics, it may be necessary to combine information from sequential acquisitions to improve CNR or SNR. The most straightforward method of combining multiple acquisitions is averaging, which improves the contrast to a noisy background as $\sqrt{N}$ for N members of the ensemble. Averaging requires a stationary object and is therefore particularly suited for high pulse repetition frequency, and low pulse energy sources, such as diode lasers (DLs) and light-emitting diodes (LEDs).^77^^–^^80^ A moving average is commonly implemented on ultrasound systems as a function called “persistence,”

A more powerful technique, singular value decomposition (SVD) filtering, can be applied to isolate stable and variable features in the image. A stationary image with variable noise can be cleaned by removal of the singular vectors that are associated with noise. Specific noise sources, artifact patterns, or laser pulse fluctuations can be removed by filtering specific singular vectors.^81^^,^^82^ Often, combinations of various filtering techniques, including SVD but also wavelet filtering, can be applied advantageously.^83^ SVD is commonly used to discriminate between flow and stationary objects, for instance, to locate hemorrhage adjacent to a major artery,^84^ to remove stationary features before flow analysis,^31^ or guide multispectral analysis in flow imaging,^82^ or in imaging of moving targets.^85^

### 2.6 Spectral Unmixing

In practice, the measured sample may contain multiple chromophores, each with its spatial distribution and molar concentration $C_{i}$. The total absorption coefficient can then be written

$$\mu_{t}(x,\lambda )=\sum_{i}C_{i}(x)\varepsilon_{i}(\lambda ),$$ (17)

where $\varepsilon_{i}(\lambda )$ is the molar absorption coefficient.^29^ Consequently, the acquired PA spectrum Eq. (10) is a composition of PA spectra acquired from this assembly of N chromophores:

$$p_{0}(x,\lambda )=\Gamma [\overset{N}{\underset{i=1}{\sum }}\varepsilon_{i}(\lambda )C_{i}(x)]F.$$ (18)

We ignore the spatial dependence of Γ and both the spatial and spectral dependence of the fluence distribution F for now. We rewrite Eq. (18) in a matrix form:

$$p_{0}(x,\lambda )=\Gamma F\cdot \varepsilon (\lambda )C^{T}(x).$$ (19)

Spectral unmixing algorithms aim to find the vector of concentrations C(x) from the estimated initial pressure distribution $p_{0}$ acquired at multiple light wavelengths  λ. Depending on the prior knowledge of the expected chromophores to be unmixed and the availability of the molar absorption spectra ε(λ), the vector C at each pixel can be found either with supervised (ε(λ) is known) or blind (ε(λ) is unknown) spectral unmixing algorithms.^86^^,^^87^

**Fig. 6:**
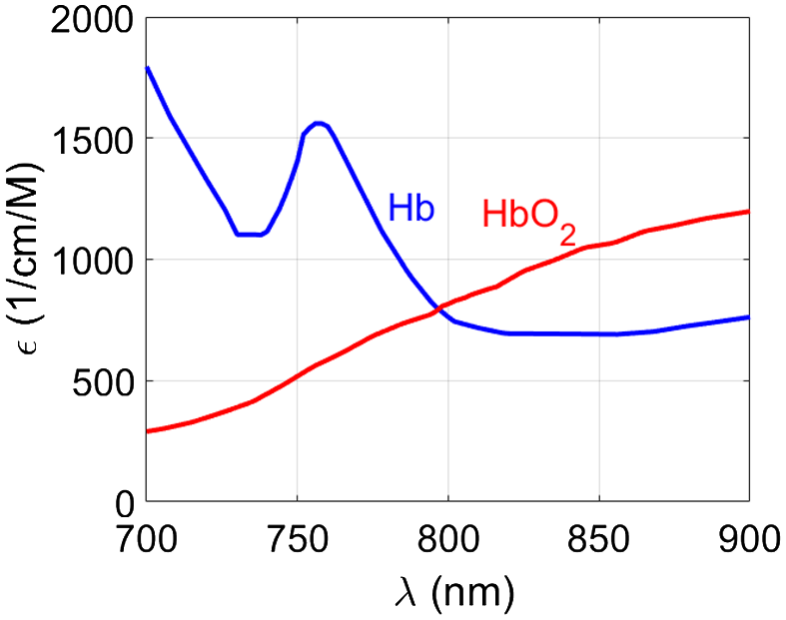
Molar extinction coefficient of oxygenated hemoglobin (${\mathrm{HbO}}_{2}$) and non-oxygenated hemoglobin (Hb).^33^ The data are available from Ref. 37.

In supervised unmixing, linear spectral unmixing is a commonly used method aiming to find the vector $C(x^{\prime })$ that minimizes the difference $p_{0}-\Gamma F\varepsilon C^{T}$. A special case is the quantification of oxygenated hemoglobin ${\mathrm{HbO}}_{2}$ and non-oxygenated hemoglobin Hb (see Fig. 6) within a blood sample using only the light two wavelengths. The relative concentrations $C_{\mathrm{Hb}}$ and $C_{\mathrm{HbO}2}$ at the chromophore’s location x can be simply found (with μ=Cε),

$$(\begin{array}{c} C_{b}(x)\\ C_{\mathrm{HbO}2}(x) \end{array})=(\begin{array}{c} \mu_{\mathrm{Hb}}(x,\lambda_{1})\;\mu_{\mathrm{HbO}2}(x,\lambda_{2})\\ \mu_{\mathrm{Hb}}(x,\lambda_{1})\;\mu_{\mathrm{HbO}2}(x,\lambda_{2}) \end{array}{)}^{-1}(\begin{array}{c} p_{0}(x,\lambda_{1})\\ p_{0}(x,\lambda_{2}) \end{array}),$$ (20)

and can be used, under the assumption of spectrally uniform fluence, to calculate the clinically relevant oxygen saturation ${\mathrm{sO}}_{2}$:

$${\mathrm{sO}}_{2}=\frac{C_{\mathrm{HbO}2}}{C_{\mathrm{HbO}2}+C_{\mathrm{Hb}}}\cdot 100\%.$$ (21)

If more wavelengths are measured than there are chromophores to be unmixed, the system becomes overdetermined and can be solved in the least-squares sense.^88^

On the other hand, unsupervised spectral unmixing methods do not require any prior knowledge of the target or background spectra. Fully unsupervised unmixing methods derive the basis set of absorption spectra from the data, which can be interesting for performing spectroscopy on biological targets. So-called “blind” unmixing methods rely on clustering of co-occurring spectral features or analyzing spectral variance throughout a data set. Examples include principle component analysis,^89^^,^^90^ independent component analysis (ICA),^91^ and non-negative matrix factorization.^92^ While unsupervised methods allow the analysis of unknown spectra, they are more sensitive to noise and other limitations of the source data as has also been demonstrated in Ref. 86.

Applying optical attenuation compensation, which can be wavelength dependent, on the reconstructed initial pressure distribution $p_{0}$ prior to spectral unmixing can substantially improve the accuracy of the estimated C.^86^^,^^87^ Fluence compensation obviously does require knowledge of the optical parameters of the sample, and the application of optical forward models as discussed in Sec. 2.2. For instance, an advanced fluence correction step is implemented in the superpixel spectral unmixing frameworks^93^ that utilizes ultrasound echo-mode images to segment tissues of different optical properties and then applies a Monte Carlo method to simulate light propagation through the sample.

### 2.7 Imaging Artifacts

#### 2.7.1 Limited view and limited bandwidth

PA waves are detected by ultrasound transducers with a broad but finite frequency bandwidth and with a limited aperture. The most prominent artifacts in PA images originate in the reconstruction of images with only a part of the emitted wave. For a complete quantitative reconstruction, the entire emitted wave must be sampled. Reconstructions with partial wavefields lead to a specific set of artifacts, which we discuss in this section. A highly instructive tutorial paper by Déan-Ben and Razansky^94^ expertly demonstrates how the interplay between absorber size and density, as well as transducer bandwidth and aperture, affect the reconstruction of PA sources.

**Fig. 7:**
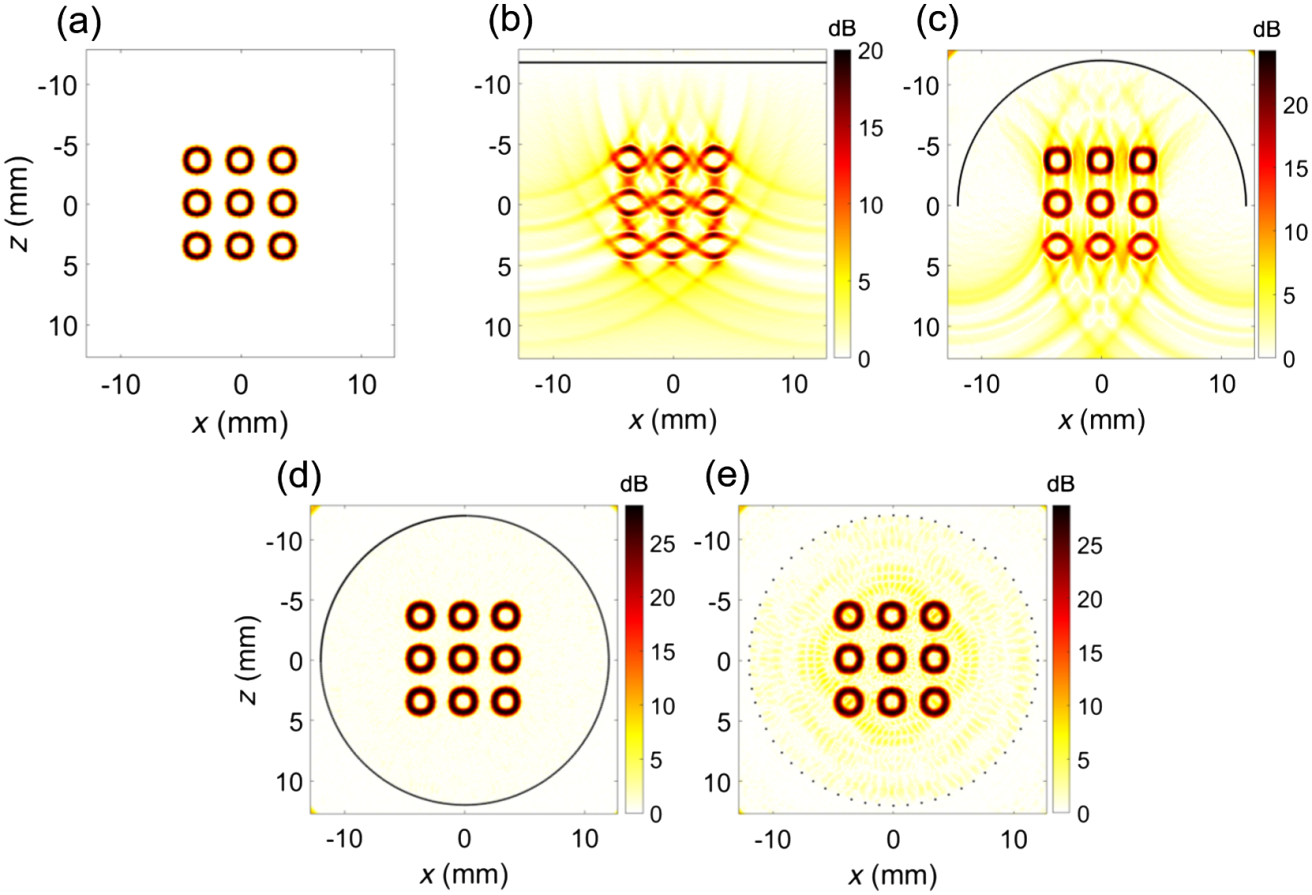
Simulated photoacoustic tomographic images of (a) a nine-ring phantom with different transducers, (b) linear with 256 elements, (c) curved with 256 elements, (d) cylindrical with 512 elements, and (e) cylindrical with 64 elements. The transducers are depicted with bold black lines. The reconstructions were performed using time reversal reconstruction. The figure demonstrates imaging artifacts appearing in different configurations such as (b) limited view artifact, (c) comet tail, and (e) sidelobe artifact.

We first take a look at aperture limitations. Waves that are not received by the transducer are not available for reconstruction. In the case of detection with a linear array, this means that waves that travel parallel to the transducer surface remain undetected and only the top and bottom but not the “sides” of a source can be visualized [see Fig. 7(b)]. Similarly, “comet tails” appear [Figs. 7(b) and 7(c)] where the cancellation of waves in the reconstruction is incomplete due to the aperture limitation. If combined PA and ultrasound imaging is used, as is frequently the case, echo features may be used to aid the reconstruction.^95^ Full 360-deg detection resolves limited-view artifacts [Fig. 7(d)]. Realistic data acquisition hardware features a limited number of acquisition channels, producing distributed artifacts as a result of degeneracy of the channel delays [Fig. 7(e)]; known as side lobes or grating lobes in ultrasound imaging.

**Fig. 8:**
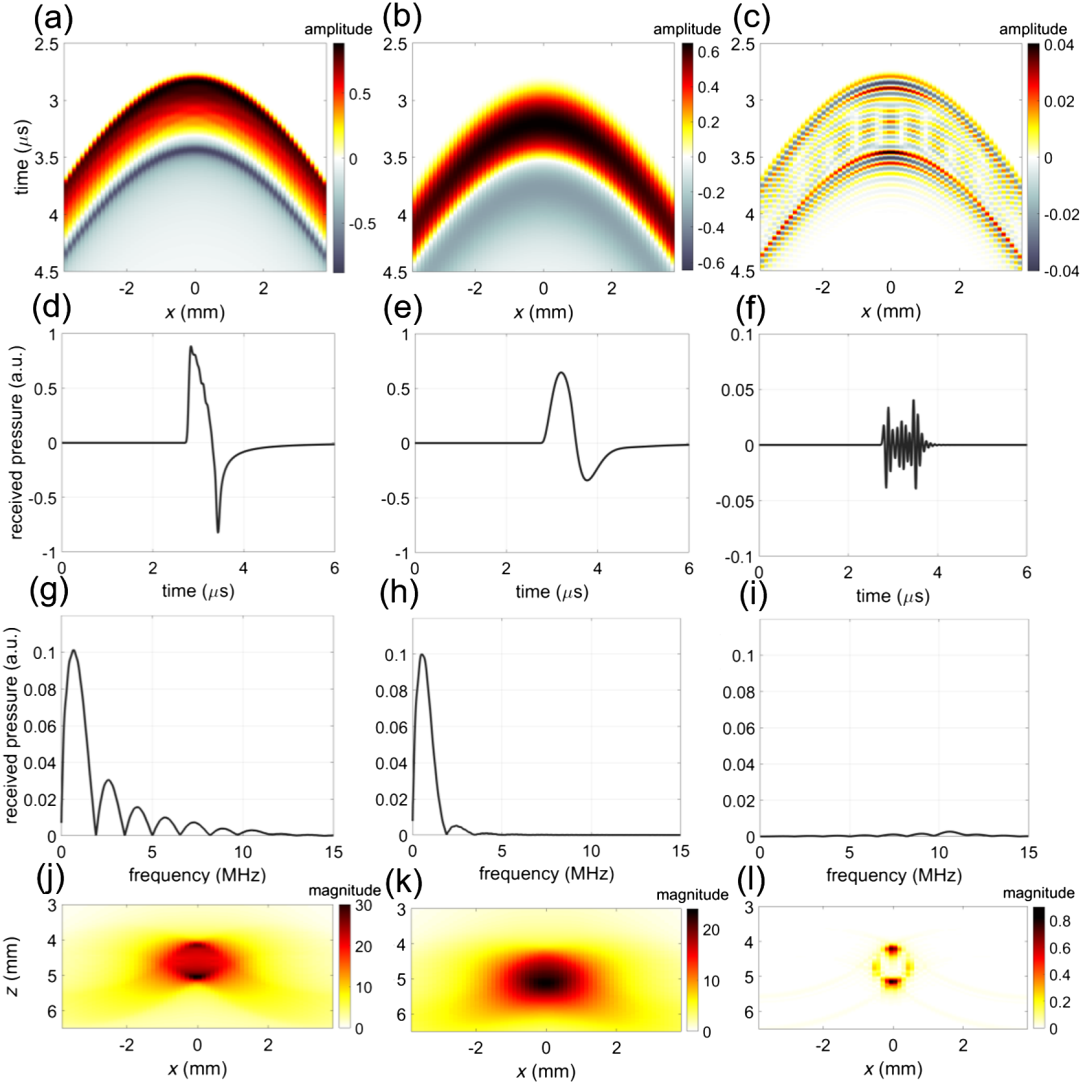
Boundary build-up effect demonstrated on a simulated pressure wave emitted by a cylindrical absorber of ø 1 mm. (a) The pressure wave was received by an (a) infinite, (b) low, and (c) high frequency ultrasound linear transducer. (d) Received pressure at the middle transducer element (x=0), (g) frequency spectrum, and (j) beamformed signal for the infinite frequency band ultrasound transducer. (e) Received pressure at the middle element, (h) frequency spectrum, and (k) beamformed signal for the low frequency band ultrasound transducer. (f) Received pressure at the middle element, (i) frequency spectrum, and (l) beamformed signal for the high frequency band ultrasound transducer. In (l), only the boundary of a large source can be visualized. The intensity of the received signal in (f) is substantially lower than in (d) and (e).

Similarly, missing frequencies as a result of finite transducer bandwidth lead to specific artifacts that are prominent in PA images. Consider, for instance, the signal emitted by a sphere of various sizes in Fig. 4. A receiving transducer with a sensitivity in the range of 2 to 5 MHz will capture only a very small fraction of the wave emitted by the smallest target, leading to a larger appearance of the target. This is analogous to the normal band limitation that also affects ultrasound imaging. That same transducer will, however, also miss the main frequency lobe of the signal emitted by the largest sphere, meaning that the lowest frequency will not be represented in the reconstruction and only the edges of the sphere are visible. This latter effect has been called the “boundary build-up” effect^96^ and is simulated in Fig. 8. It is frequently observed in blood vessels.^97^ Frequency compounding can be applied to faithfully reconstruct targets with a range of sizes.^98^

Missing features, boundary build-up, sidelobes, and other artifacts have been remedied using deep learning methods and have been extensively reviewed.^99^^–^^102^ Very briefly, reconstruction networks are trained on known structures, from which PA signals can be computed using optical and acoustic forward models, combined with the instrument response. The application of the reconstruction network to experimental data then yields images with suppressed artifacts and more accurate quantification.^103^^,^^104^

Artifacts that originate in the partial reception of the PA wave, either in frequency or in direction, can be mitigated at least partially by changes to the transducer and data acquisition configuration. Several experimental innovations in transduction technology, such as the use of concave arrays, optical detection, and non-resonant detectors, aim to extend the information captured for image reconstruction. This topic merits its own discussion (see Sec. 3.1).

#### 2.7.2 Spectral coloring

As light traverses the tissue, it is subject to attenuation due to scattering and absorption. Many PAI applications use transcutaneous illumination, where the optical illumination passes through the skin and the superficial tissue optics affect the optical power delivered to deeper-lying imaging targets. Since the skin contains various heterogeneously distributed chromophores, such as blood, pigment, and collagen, it does so in a wavelength-dependent manner. The resulting change in the spectral content of the excitation light affects the relative response of a known chromophore at different wavelengths, which complicates spectral unmixing. For interstitial illumination, the skin can be bypassed, but similar arguments apply to internal tissues such as mucous membranes.

Optical attenuation is the cumulative sum of scattering and absorption. The use of generic tissue can mimic real tissue and expected optical properties can be calculated;^105^ however, chromophore concentrations and compositions vary substantially from person to person. Scattering in human tissue varies only weakly with wavelength throughout the near-infrared (NIR; 750 to 1400 nm), extending into the short-wave infrared (SWIR) spectral region up to 2  μm.^39^ Skin has a larger scattering coefficient than adipose or mucous tissue, but in all tissue types, the spectral variation is smooth and can be approximated by a power law $\mu_{s}^{\prime }∼\lambda^{-y}$. This means that spectral coloring due to scattering is limited and can usually be neglected.

The absolute scattering coefficient does vary between subjects and depends on skin type and skin thickness. Pigmented skin with higher melanin concentrations exhibits stronger scattering,^106^ impairing penetration depth. Skin thickness depends on the location of the body.^107^ Particularly, the hypodermal layer, which mainly consists of adipose tissue, varies in thickness.^108^

Depending on the chromophore type, distribution, and concentration in the tissue layers that are in between the light source and the imaging target, the local absorption spectrum changes the spectral content of the illuminating fluence. Especially, the skin melanin concentration should be considered^109^ since it induces spectral coloring that leads to underestimation of oxygen saturation in subjects with darker skin types. The difference in skin tone, often classified with the Fitzpatrick scale,^110^ has been often overlooked or even disregarded in PAI research. Since biological readings are affected by superficial melanin, it is important to develop accurate, spectrally resolved correction factors.

Besides skin melanin, the blood in superficial vessels and capillaries induces spectral coloring, depending on its volume and oxygenation state. Differential absorption between the wavelengths used to determine oxygen saturation deep in tissue can strongly affect the analysis.^111^ The selection of wavelengths that minimizes this error is non-trivial: somewhat counterintuitively, large absorption contrast is best avoided since this leads to a strong impact of spectral coloring that can only be compensated for if there is sufficient signal remaining, and a suitable tissue optical model can be formulated.

For wavelengths greater than ∼900  nm, water absorption should be accounted for when estimating spectral fluence, in addition to the biological chromophores we discussed above. Adipose tissue exhibits strong absorption bands near 1.2 and 1.7  μm.

Spectral coloring can be accounted for in spectral unmixing by explicitly including the wavelength dependence of the fluence as a diagonal matrix term in Eq. (18):

$$p_{0}(x,\lambda )=\Gamma [\overset{N}{\underset{i=1}{\sum }}\varepsilon_{i}(\lambda )C_{i}(x)]F(x,\lambda ).$$ (22)

Given a model of the distribution of the relevant chromophores, F(x,λ) can be computed using a suitable optical forward model.^111^

#### 2.7.3 Clutter

The diffuse fluence distribution resulting from the illumination of scattering tissues delivers optical energy to a volume of tissue that is generally not confined to the imaging field of view. Out-of-plane absorption and reflections of the generated PA waves commonly generate so-called clutter artifacts in images, and spurious image features that do not coincide with an actual absorber in the image. Clutter can be a serious problem in PA image interpretation, introducing features where there are none in reality or masking weak features by strong clutter signals. In interpreting PA images, it is important to understand the mechanisms that cause these features and to recognize them.

**Fig. 9:**
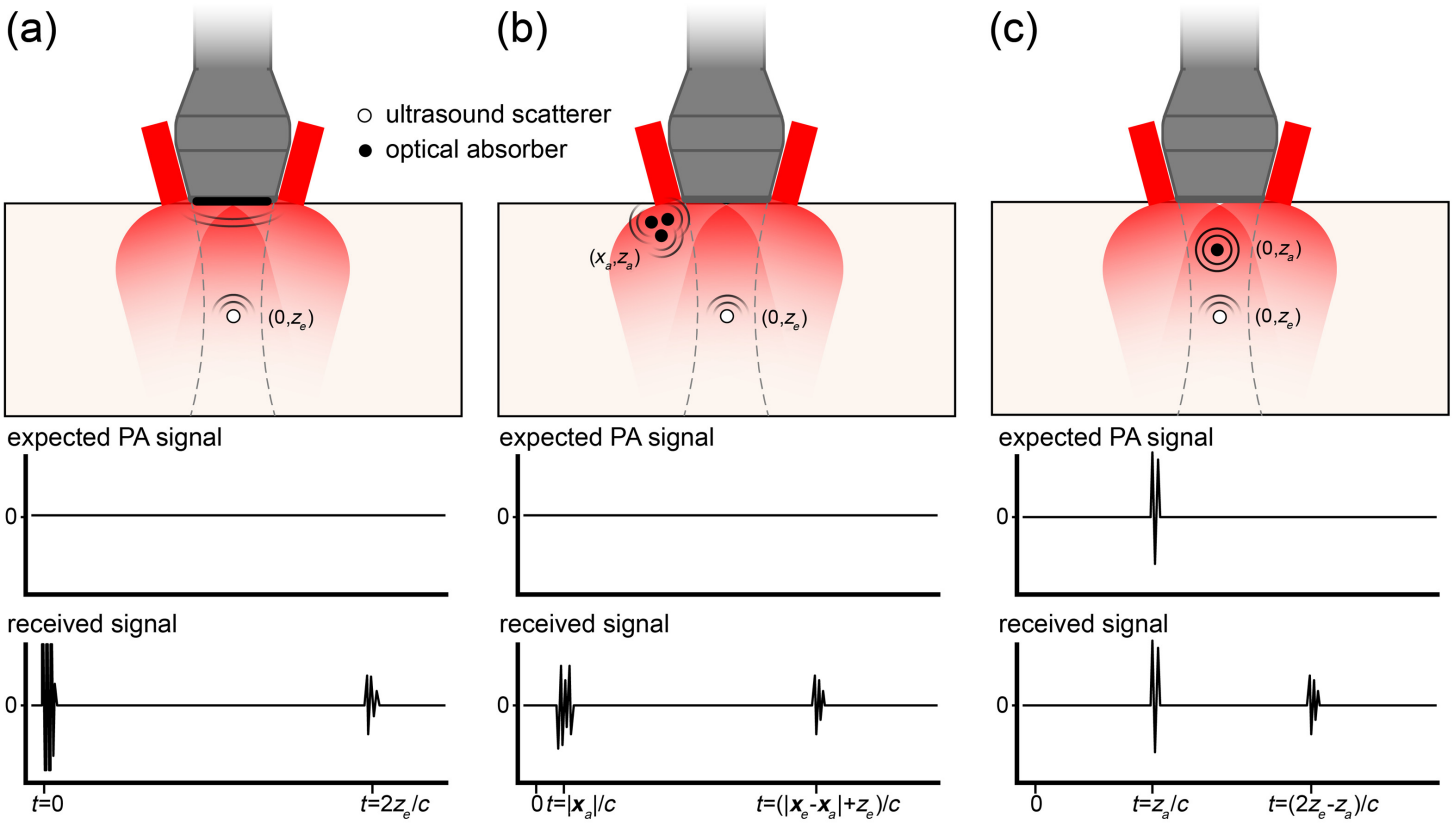
Mechanisms producing image clutter, illustrated in a dual-sided illumination geometry. Dashed lines indicate the borders of the image plane, which is perpendicular to the page. (a) Light absorption in the transducer surface generates a strong signal at the moment of laser pulse emission (t=0), and an acoustic wave that can be reflected by a scattering object at xe=(0,ze). (b) Superficial absorbers located at xa, outside the transducer field of view but within the illuminated area may receive a high fluence and emit a strong PA signal, which can reach the probe directly, and can be reflected off a scattering object at xe in the image plane. (c) PA signals generated by an absorbing target in the imaging plane may be reflected by ultrasound scatterers.

Figure 9 summarizes the three most significant sources of image clutter, illustrated in a common geometry of illumination from the sides of the ultrasound probe. Strong signals can arise from optical absorption in the transducer front surface itself, sketched in Fig. 9(a). The thermoelastic response in the lens or matching layer covering the active layer in the transducer will generate a direct electric signal. This signal is frequently strong enough to saturate the receive amplifiers of the data acquisition system. The transducer surface will emit an optically generated ultrasound wave, which can reflect off any ultrasound scatterers in the image and create an echo. The application of a white front layer of the ultrasound probe is a simple measure that can partially mitigate this effect.

Similarly, superficial absorbers directly in the optical delivery beam [Fig. 9(b)] can create strong PA signals that can be detected by the probe, either directly or via ultrasound reflection.^112^ Like PA generation in the transducer, even weak absorption can give rise to a strong signal because of the very high local fluence that is present at the surface, outside the imaging plane in this particular geometry. Superficial vasculature or melanin in the skin can produce image clutter by this mechanism.

Finally, PA signals that originate from targets that are inside the field of view may be reflected by ultrasound scatterers, producing an echo [Fig. 9(c)]. All these artifacts need to be minimized if possible and otherwise considered in image interpretation. Methods to correct clutter have been proposed based on motion tracking.^112^^,^^113^

The amount and appearance of clutter in the PA image is strongly affected by the relative positioning of the illumination relative to the tissue and the transducer. Moving the light delivery away from the transducer can suppress strong surface-related signals. If that is not possible or the effect is insufficient, clutter can be recognized during image interpretation by its characteristic delay time signature, as sketched in Fig. 9. The use of a collocated echo image can greatly facilitate the identification of acoustic reflectors in the field of view.

## 3 Instrumentation

Functionally, a PAI system consists of a light source, a light delivery system, ultrasound transduction, amplification, filtering and other signal conditioning, analog-to-digital conversion, and finally an image reconstruction, processing, and display unit. In the practical settings that we focus on in this review, PAI is combined with echo imaging in the majority of studies. This means that ultrasound transmission is an important requirement, adding pulse generation and transmit switching to the functional system diagram. The requirements of all components of a PAI system are dictated by the application requirements, such as imaging volume, resolution, contrast, and speed.

By applying a broad optical illumination and using multi-element arrays, with parallel capture of PA signals, PAT enables the reconstruction of larger two-dimensional (2D) or three-dimensional (3D) imaging regions for each laser pulse. The ultrasonic detection determines the spatial resolution, where a lower acoustic frequency leads to an increase in the imaging depth. Different PAT configurations, transducers, and light sources offer a wide variety of strategies, each with its own considerations.

In the case of photoacoustic microscopy (PAM), the light and the acoustic detection are focused on a small area, resulting in a high spatial resolution but relatively low imaging depth and speed. When the optical focus is smaller than the acoustic focus, it is referred to as optical-resolution PAM. Conversely, when the acoustic focus is smaller than the illuminated area, it is called acoustic-resolution PAM. Using a single element transducer, each laser pulse generates one image line, or even a single image pixel in optical-resolution PAM. With 2D raster scanning, PA images can be acquired. As clinical applications of PAM have been limited to date, we refer interested readers to dedicated reviews for information about different PAM configurations for specific applications, such as microangiography and functional imaging.^114^^–^^116^ One notable exception is raster-scanning optoacoustic mesoscopy (RSOM), which has been applied in dermatology.^117^ PA endoscopy may be considered as a miniaturized form of PAM for invasive imaging. Most endoscopes apply rotational scanning on probes that enter the colon,^118^^–^^120^ esophagus,^118^ or coronary arteries.^121^^–^^123^ The acquired A-lines are often displayed in a B-mode image in polar coordinates.

### 3.1 Ultrasound Detection

#### 3.1.1 Transducers

The ultrasound transducer is an essential component of a PAI system, which converts mechanical energy into electrical energy. Very often, piezoelectric ceramics or crystals are used for this purpose, which vibrate as a result of the mechanical PA wave, leading to the production of electrical signals in the connected electrodes. In the case of echography, the ultrasound wave is transmitted by applying an electrical pulse to the transducer. The PA signals are processed by the PAI system to generate images, as discussed in Sec. 2.5. The choice of shape, size, and frequency is tailored to the specific imaging needs.

Linear array ultrasound transducers are widely used in daily clinical practice and therefore commonly used in PAT. They are affordable, handheld operable, and PA acquisition could be integrated with conventional ultrasound systems. However, due to the omnidirectional origin of the PA waves the linear array transducer suffers from a limited view problem, discussed in Sec. 2.7.1. Accordingly, it misses signals originating from the sides of the PA sources and thus impairs image reconstruction and causes artifacts, as shown in Fig. 7.^124^ Different imaging methods, such as rotational imaging^125^^,^^126^ and dual transducer imaging schemes,^127^^,^^128^ were developed to mitigate this effect. While these imaging configurations enhance image quality under controlled laboratory conditions, their usefulness in a clinical setting is restricted by practical limitations.

Concave array transducers were designed to overcome the limited aperture by covering a larger angle of view of the wavefront. Arc-shaped transducers have a geometric shape that results in a natural focus, increasing the aperture.^129^ Ring-shaped transducers cover the full 360 deg and thus are even less influenced by the directionality of the PA waves.^130^^,^^131^ Hemispherical detectors can receive 3D PA signals and have been developed as a handheld “cup” transducer.^66^ Because of the geometrical focus of curved array transducers, there is an anisotropic spatial resolution with a higher resolution within the ultrasound focal area. Curved array transducers offer advantages over linear array transducers but are more complicated in design, expensive, and require specific designs for different applications due to the relatively smaller imaging area. Especially, the ring-shaped and hemispherical transducers require much more space, which limits the clinical applicability.

Conventional ultrasound transducers are built for specific applications based on imaging depth, field of view, and resolution requirements. In general, these transducers have a narrow bandwidth. This results in the partial acquisition of the full frequency spectrum, as discussed in Sec. 2.7.1. Since laser power is not only expensive but also limited, it is of great importance to pursue receive sensitivity and thus a sufficient amount of the generated acoustic wave. To limit the rejection of out-of-band signals, the center frequency and bandwidth of the ultrasound transducer should be ideally matched to the PAI target. To increase the sensitivity to the broadband PA signals, different custom broad-bandwidth transducers have been demonstrated *in vivo* for specific applications, such as psoriasis, breast, and brain imaging.^117^^,^^132^^,^^133^ Also multiple transducers with a complementary bandwidth have been applied to cover a wider spectral range.^134^^,^^135^ To combine data from different ultrasound probes, relative transducer sensitivities should be taken into account.^98^

An alternative technology to piezoelectrics, which offers a wider bandwidth, is the capacitive micromachined ultrasound transducer (CMUT). Using a capacitor cell with attached electrodes on a fixed plate and a flexible membrane, changes in capacitance due to vibrations in the flexible membrane can be detected.^136^ In addition to greater bandwidth, CMUTs offer improvements in SNR and CNR.^137^ All-optical transducers, such as Fabry–Pérot interferometric sensors, also offer a broad bandwidth reception and can be produced in small sizes without compromising sensitivity, which makes them suitable for endoscopic applications.^138^^–^^140^

For practical PAI, the combination with ultrasound echography is almost indispensable. Interferometric sensors cannot transmit ultrasound and so need separate acoustic emitters for echo imaging. Likewise, some piezoelectric transducers based on poly-vinylidene difluoride or other polymers are not suitable for transmission due to their impedance characteristics but can be configured for efficient reception of ultrasound signals.^22^^,^^141^

#### 3.1.2 Data acquisition

The received PA signals usually undergo several signal conditioning steps, in the so-called analog front end (AFE) of the ultrasound system, prior to digitization. Bandpass filtering removes noise and spurious signals. Subsequently, the signals are amplified and converted to digital signals before further processing for image reconstruction and storage. TGC selectively amplifies deep (weaker) signals and can also suppress strong signals, for instance those originating from the surface. In programmable research ultrasound systems,^142^^–^^144^ these filtering and amplification steps can be customized with considerable freedom, and optimization for PA acquisition in specific applications is possible. Important factors determining image quality are the AFE noise, bandwidth, and dynamic range. Ultrasound systems can feature a usable dynamic range of up to 100 dB. For commercial, clinical, ultrasound systems, details of the AFE and digital signal processing are usually proprietary. These are extremely sophisticated signal processing systems that are highly optimized to their primary function, which is pulse-echo imaging, and the flexibility for PAI is usually limited.

To perform interleaved ultrasound echography using the same transducer elements, it is necessary to synchronize the switching of the channels transmit or receive with the optical illumination pulses. While echo systems offer considerable dynamic range, some dedicated PA systems use a high-impedance receive system, which enhances sensitivity but precludes ultrasound transmission, and so separate signal acquisition subsystems are needed for PAI and ultrasound.^67^

The channel count is possibly the most important property of the acquisition system since this dictates the number of transducer elements that can be sampled and processed simultaneously. More receive channels allow for better spatial resolution, field of view, and SNR that can be collected from each laser pulse. Since the optical power is costly and restricted to prevent tissue damage, and high-power laser systems can have a low pulse rate, parallel digitization of as many channels as reasonably possible is essential. The number of channels is limited due to the high cost of the equipment, complexity of the system, and amount of data transfer and processing power required. Therefore, there is a tradeoff between image quality and system complexity and cost. The ability to store and access channel RF data, prior to image reconstruction is another important concern. Programmable clinical ultrasound systems have been used for PAI.^145^^–^^147^

### 3.2 Light Sources and Illumination

The short light pulses required to achieve stress confinement, which is a condition for high-contrast, high-resolution PAI with optimal sensitivity, put stringent demands on the light sources used to generate the signal. The wavelengths that provide the optimal contrast for imaging a particular biological property are dictated by the absorption spectra of endogenous chromophores of interest (Fig. 2), which do not necessarily coincide with the wavelengths provided by common laser gain media such as Nd-doped yttrium aluminum garnet (Nd:YAG; 1064 nm) or Er-doped glass fiber (around 1560 nm). That said, both Nd:YAG and Alexandrite lasers have been usefully deployed for breast imaging.^148^^–^^150^ If exogenous contrast can be used, there is a little more freedom since the absorbing agent can be designed or chosen for accessible or contrasting wavelengths. Even with the use of exogenous contrast, however, tissue optical properties that cause optical attenuation specify windows in which useful contrast can be attained, as discussed in Sec. 2.7.2. In particular, water, hemoglobin, and melanin absorption can be limiting factors, in combination with the scattering coefficient, which generally increases for shorter wavelengths.

Optical attenuation rapidly reduces the fluence as light propagates into scattering tissue, which means that incident power is often the limiting factor in image penetration depth at a given ultrasound detection sensitivity. At the same time, exposure to optical radiation is limited by safety concerns, setting a maximum to the pulse energy that can be applied, depending on illumination configuration, pulse frequency, and wavelength.

#### 3.2.1 Tunable solid-state laser systems

The desire for a combination of wavelength flexibility and high pulse energy has led to the popularity of tunable solid-state lasers, usually based on a Q-switched rare-earth-doped crystal (such as Nd:YAG) laser. The energy storage capacity allowed by Q-switched laser cavities allows very large pulse energies (100s of mJ to about 1 J) to be generated in pulses with a typical duration of 10 ns and wavelengths near 1  μm.

The very high peak power of Q-switched Nd:YAG lasers, which can range up to 100 MW, also allows for the pumping of optical parametric oscillators (OPOs), which offer unparalleled wavelength flexibility. In practice, these systems are often pumped by the second (532 nm) or third (355 nm) harmonic, and several of such systems are offered commercially, specifically for PAI. Even though parametric generation is not a very efficient process, these laser systems can still produce tunable output in the order of 50 mJ per pulse or more. The pulse duration $t_{p}$ is comparable to the pump pulse width, in the order of 3 to 30 ns. The pulse frequency of these systems is commonly between 10 and 200 Hz; state-of-the-art systems allow pulse-to-pulse wavelength switching, which is attractive for spectroscopic imaging. OPOs produce a signal (shorter) and idler (longer than double the pump wavelength) beam. The shortest wavelengths that can be produced are typically around 420 nm for OPOs pumped with 355 nm light (the third harmonic of Nd:YAG) and around 660 nm when pumped with 532 nm light (second harmonic of Nd:YAG). The longest wavelengths that can be generated are about 2.5  μm, where quartz optics become absorbing.^151^ The highest pulse energy is normally generated in the short-wavelength spectral range of the signal beam. These broadband tunable OPO systems often have a suboptimal beam quality, with beam quality factors up to $M^{2}\approx 50$. This can be a limiting factor for coupling to small fibers or creating a small focus but works well for broad area illumination or coupling to a fiber bundle.

High-power laser systems, and specifically OPO systems, tend to be expensive, large, and require water cooling, which can be noisy. These properties make integration in commercial mobile units less attractive. Lower-energy Q-switched lasers with pulse rates of several kHz, fitted with a wavelength conversion mechanism, have also been used. These are generally configured with less wavelength flexibility and have proved their value in scanning-probe imaging.^152^^,^^153^ In PAM, frequency-doubled Nd:YAG pumped dye lasers have been used to achieve custom wavelengths,^154^ but fluid management and spill risk make these impractical for use in a clinical setting. Alternatively, lasers based on wavelength shifting by stimulated Raman scattering (SRS) in silica fiber provide a promising outlook.^19^^,^^155^^,^^156^ These lasers offer a high (sometimes even single-mode) beam quality, but pulse energies remain too low for PAT at present.

#### 3.2.2 Semiconductor lasers and light-emitting diodes

The drawbacks of size, cooling requirements, and noise, associated with high-power solid-state lasers, can be overcome by the adoption of semiconductor sources. These exist in a fairly broad range of fixed wavelengths in the visible and NIR spectral range. Some of those fortuitously coincide with spectral features of biological interest, such as the common laser diode wavelength of 808 nm close to the isosbestic point of HbO_2_ and Hb and to the absorption maximum of albumin-bound indocyanine green (ICG).^157^

Both DLs^77^^,^^79^^,^^158^ and LEDs^78^^,^^80^ have been used for PAI. Semiconductor lasers or LEDs can be operated in pulsed mode by modulating the drive current, which offers flexibility in shaping the output pulse, for instance, to match the frequency response of the transducer.^159^ The main limitation of most semiconductor sources is the lower energy density of the gain medium: as a result, common pulse energies are lower by up to three orders of magnitude compared to Q-switched lasers. Larger pulse energy can be achieved by lengthening to pulse in time, but this is limited by stress confinement as discussed in Sec. 2.1.^79^ By stacking diode arrays in a compact driver-diode assembly, larger pulse energies can be achieved, albeit at the cost of beam quality.^160^^,^^161^

A common strategy to enhance the SNR in recordings made with low pulse energy has been to average multiple traces,^77^ increasing the SNR in proportion with the square root of the number of acquisitions. The pulse rate of semiconductor sources is limited in practice by thermal considerations, and by the acoustic delay of the PA signal, resulting in pulse rates of several, up to 10 kHz. If imaging frame rates of at least a few Hz are desired, the SNR gain that can be achieved by averaging is limited to a factor of 30, approximately. Coded excitation is a more sophisticated method to combine the energy of multiple pulses. Using either frequency modulation,^162^ digital codes,^78^^,^^163^ or frequency-coded pulse trains,^20^ the time-domain PA signal can be reconstructed from the received pressure trace by decoding using filtering or Fourier transformation.

#### 3.2.3 Fiber lasers

Rare-earth doped fiber lasers offer an as yet underexplored but possibly attractive alternative to solid-state lasers or semiconductor sources. In a fiber amplifier, a seed pulse from a DL or superluminescent diode is amplified by one or multiple amplifier stages, which are again pumped by DLs, typically at 980 nm. Fiber lasers are more compact than solid-state lasers, require no or very little alignment, providing stability and robustness, and can be air-cooled. Compared with semiconductor sources, fiber lasers exhibit higher output while offering similar flexibility in pulse duration and frequency.^164^ Both Yb-doped (accessible wavelengths 1030 to 1080 nm) and Er-doped (1530 to 1565 nm) fiber amplifiers have been used for laser systems aimed at PAI.

Many laser systems can be realized in single-mode fiber, resulting in superior beam quality. This aspect greatly facilitates nonlinear wavelength conversion by SRS or four-wave mixing to achieve specific excitation wavelengths in the SWIR spectral band.^19^^,^^165^^,^^166^

#### 3.2.4 Light delivery configurations

Since optical attenuation remains the main limiting factor in PAI, the design of the optical illumination configuration is very important to achieve optimal images. A second consideration is the appearance of imaging artifacts, which can be strongly affected by details in the optical arrangement, as discussed in Sec. 2.7.1.

**Fig. 10:**
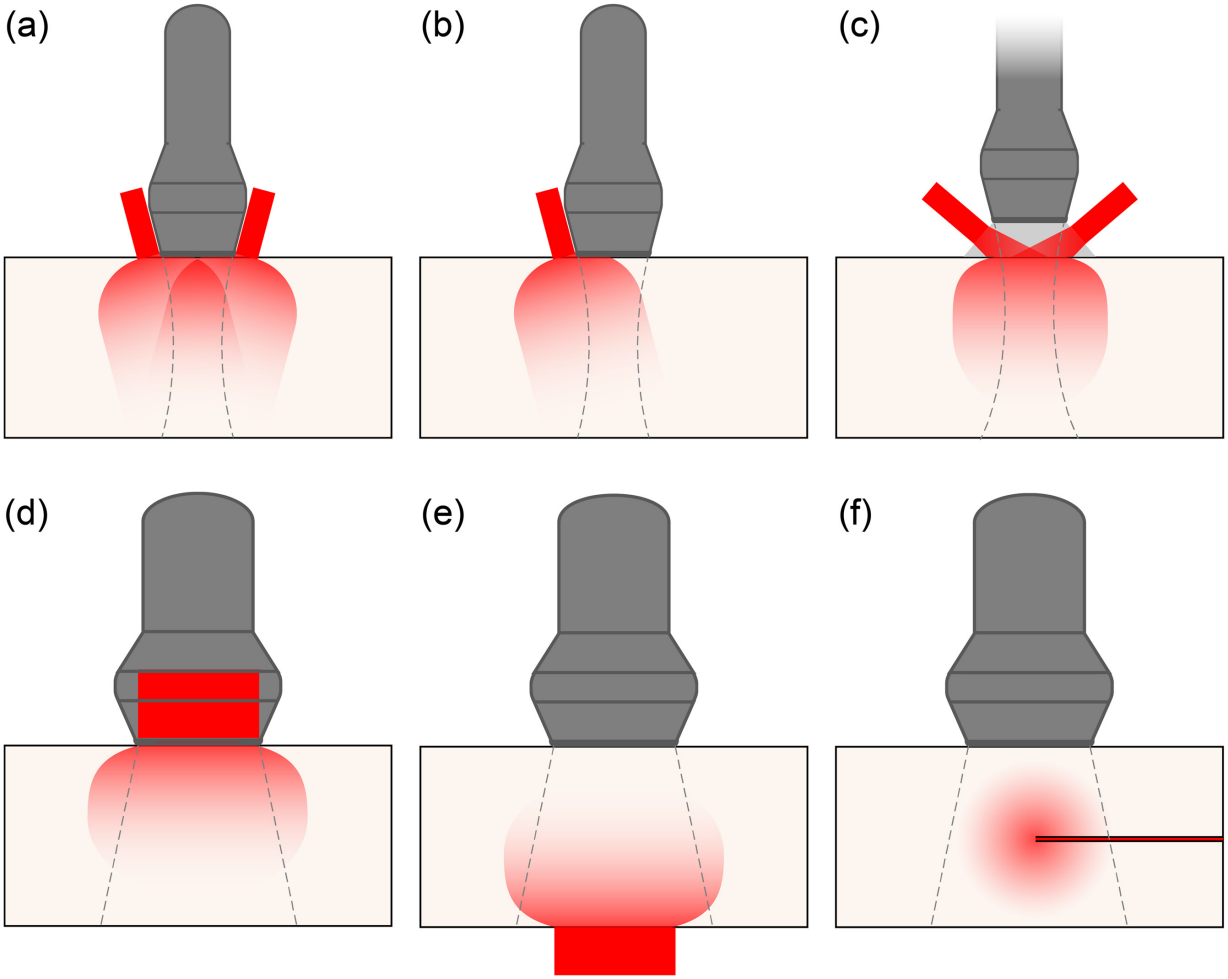
Illumination geometries. (a) Illumination from both sides of a linear array probe, producing two overlapping volumes of diffuse illumination (image is perpendicular to the page). (b) Single-sided illumination. (c) Illumination through a standoff that is both optically and acoustically transparent. (d) Illumination through a transparent transducer (image plane is parallel to the page). (e) “Transmission” geometry: light is delivered from the distal side of the anatomy under study. (f) Interstitial illumination using a fiber probe in a needle or catheter. Dashed lines indicate the approximate field of view.

In handheld tomographic imaging, a broad illumination area is usually achieved by mounting a fiber bundle on the side, or sides, of the ultrasound probe, as shown in Figs. 10(a) and 10(b). Similarly, DL or LED assemblies may be mounted on the side of the transducer.^80^^,^^160^ These configurations have the advantages of simplicity and versatility: the light moves with the ultrasound field of view and is delivered to the tissue under investigation with the aid of optical scattering in the tissue.

Illumination from the probe delivers the highest fluence at the tissue surface outside the field of view of the probe, where it may generate pronounced PA artifacts through out-of-plane signals or echoes that may complicate image interpretation.^167^ The use of a standoff made of gel that is transparent to both light and ultrasound^168^ [Fig. 10(c)] allows illumination of the probe field of view, as does through-transducer illumination^169^ [Fig. 10(d)]. Nevertheless, also in this arrangement, a small amount of surface absorption will generate a strong PA signal on the tissue interface, which may generate clutter.

Administering the light in a “transmission” geometry opposite from the ultrasound probe [Fig. 10(e)], if accessible, may be a suitable strategy for deep targets. Interstitial illumination [Fig. 10(f)] is an alternative strategy that avoids most of the mechanisms that generate clutter artifacts and circumvents much of the optical attenuation of the tissue between the ultrasound probe and the image target.^170^^–^^172^ It is suitable for imaging deep tissues that are accessible to optical fibers, either through a natural orifice, or possibly transcutaneously, if in an intervention setting. The decoupling of illumination and ultrasound probe does introduce the problem of colocalization of light delivery and ultrasound field of view.

### 3.3 Contrast Agents

Photoacoustic contrast agents (PACAs) are exogenous chromophores that can be used to create PA contrast for tissue types that do not exhibit intrinsic absorption contrast. Targeted PACAs can be designed to visualize specific tissue types by attaching a targeting ligand to an absorbing particle or molecule.^12^ The absorbing entity produces the PA response while being attached to the tissue of interest via the ligand. In this manner, highly specific images can be generated. This principle has been used to detect early metastases in lymph nodes in oral cancer patients.^173^ Alternatively, untargeted exogenous contrast may be administered, which highlight areas where the contrast agent pools, such as in dysfunctional lymphatic vessels.^168^

PACAs offer a relative freedom in choosing the wavelengths that generate contrast, and major biological chromophore absorption bands can potentially be avoided. Many absorbing (nano)particles and dyes that are used as PACAs have originally been developed as fluorescent agents and thus have been designed to re-emit part of the absorbed energy as optical radiation. For PAI, this energy does not contribute to the signal and can potentially contribute to unwanted background signal.

PACA absorbers can be broadly classified into small-molecule dyes and nanostructures, where the latter category includes metallic, organic, inorganic semiconductor, and carbon particles.^12^ Due to their larger size, nanoparticles and nanoconstructs have lower mobility, and as a result are more difficult to clear from the body. Toxicity is a greater concern for nanostructures than it is for dyes. For this reason, research on humans has been performed with small-molecule dyes only, either targeted or untargeted.

**Fig. 11:**
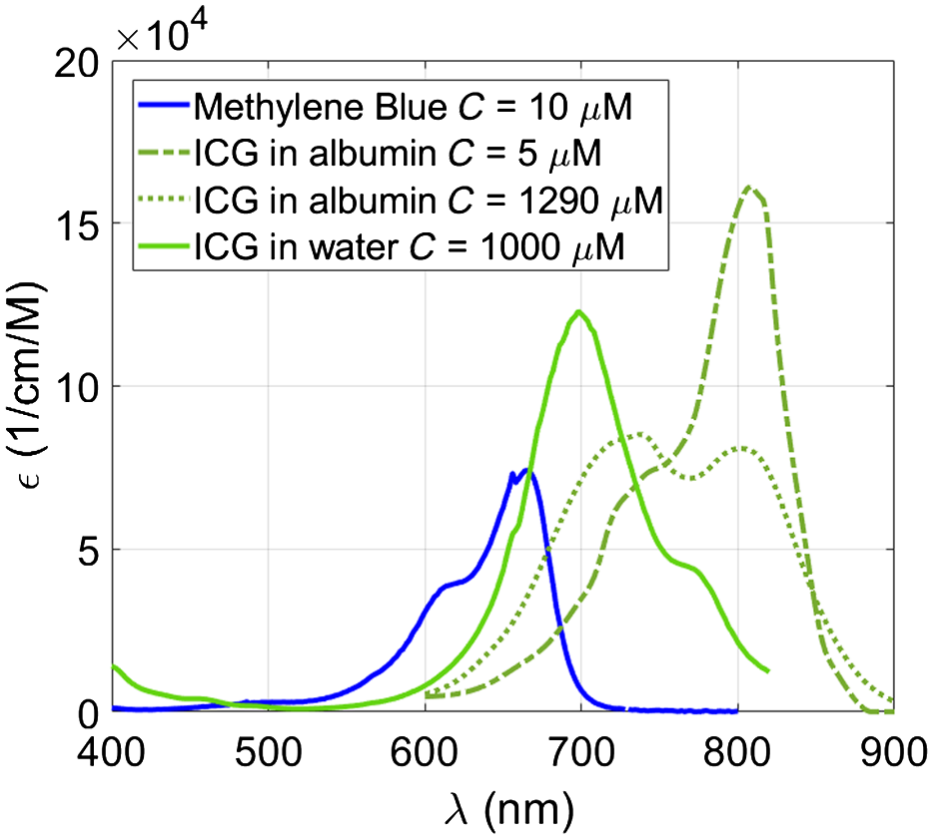
Molar extinction coefficient of methylene blue and ICG in different solvents at different concentrations. Molar extinction coefficient (C) of oxygenated hemoglobin (${\mathrm{HbO}}_{2}$) and non-oxygenated hemoglobin (Hb). Spectra are available from Ref. 37.

Among the many available dyes, Evans blue (absorption around 600 nm), methylene blue (670 nm), and ICG (800 nm) are approved for human administration by the FDA. These dyes are relatively small (<2  nm) and exhibit low toxicity, making them suitable for clinical use. The spectra of methylene blue and ICG are shown in Fig. 11.

ICG is extensively used in various clinical applications for diagnosis and surgical guidance. ICG belongs to the family of cyanine dyes, which also encompasses popular alternatives such as CW800, Cy5, and Cy7. ICG possesses both hydrophobic and lipophobic properties, making it suitable for angiography.^174^ The ICG absorption spectrum is notoriously variable and is sensitive to concentration, solvent, availability of serum albumin, temperature, and storage time (see Fig. 11).^12^^,^^157^ For instance, ICG is prone to J-aggregation, which causes the formation of a collateral absorption peak due to the clustering of ICG molecules.^175^ J-aggregation was reported also for other cyanine dyes.^176^

### 3.4 Commercial Systems Overview

This section provides an overview of the current commercial clinical systems that are used in a clinical research setting. It is important to note that this is an evolving field, and therefore this section may be subject to change as new systems emerge.

The Acuity Echo (iThera Medical GmbH, Munich, Germany) is a CE-marked, handheld PA/ultrasound system that offers further non-CE-certified customizations.^177^ The system has a tunable laser with a wavelength range of 690 to 980 mJ (optionally 660 to 1300 nm) with an adjustable repetition rate of ≤25  Hz and pulse energies up to 25 mJ at 750 nm, which enables spectral imaging. The system has an arc-shaped detector with 256 elements at a 4 MHz center frequency for 2D imaging and offers a hemispherical cup detector with 256 to 384 elements at a center frequency of 8 MHz. The system is applied in various clinical research studies in applications, such as inflammatory bowel disease,^13^ vascular malformations,^178^ and thyroid assessment.^179^

The Acoustic X (Cyberdyne Inc., Tsukuba, Japan) is a handheld, pre-clinical PA/ultrasound system that is applied in various clinical research areas, such as the detection of joint inflammation,^180^ port-wine stain imaging assessment,^181^ and lymphatic vessel imaging.^168^ It is an LED-based system with a 128-element linear array transducer with a center frequency of 7 or 10 MHz. The illumination is provided with side-illuminating high-density, high-power LED strips at fixed wavelengths of 690, 750, 820, 850, and 940 nm (combinations 690/850, 750/850, and 820/940 nm). The pulse width and pulse repetition frequency are variable between 30 and 150 ns and 1, 2, 3, or 4 kHz.

The PA system LAZR-X (FUJIFILM VisualSonics, Toronto, Canada) offers a variety of linear high frequency transducers and a wavelength range of 680 to 970 and 1200 to 2000 nm. It has been applied widely in pre-clinical research, but its use with humans has been scarce.^182^

An FDA approved, clinical handheld PA/ultrasound imaging system that is specifically developed for breast imaging is the Imagio (Seno Medical, San Antonio, Texas). Its dual-wavelength (757 and 1064 nm) approach allows for blood vessel imaging. The 128-element linear array transducer with a frequency range of 4 to 16 MHz is used for both pulse-echo and PA reception.^183^

The LOUISA-3D (TomoWave Laboratories, Inc., Houston) is another clinical dual modality PA/ultrasound breast scanner. The imaging bowl with coupling medium is illuminated by a rotating, 90 deg arc-shaped optical fiber (dual-wavelength at 757 and 797 nm, pulse energy up to 800 mJ, 10 Hz repetition rate). An additional rotating, 90 deg arc contains 96 broad bandwidth (50 kHz to 6 MHz) ultrasound transducers for the PA reception. The ultrasound imaging is based on a 90 deg arc-shaped ultrasound array with 192 elements and a center frequency of 7 MHz.^184^

Finally, a clinical, mobile acoustic-resolution PAM system designed for skin imaging is the RSOM Explorer C50 (iThera Medical GmbH, Munich, Germany), which has a 50 MHz center frequency (10 to 180 MHz) transducer and illuminates at 532 nm (pulse repetition frequency up to 500 Hz, pulse energy up to 250  μJ with a pulse width of 2.5 ns). The resolution of up to 10  μm up to several millimeters depth allows for microvasculature imaging that could be applied for diagnosis or treatment monitoring of diseases, such as psoriasis^117^ or systemic sclerosis.^185^

## 4 Safety and Standardization

### 4.1 Safety and Optical Exposure Limits

Biological tissues can be damaged by light. Maximum permissible exposure (MPE) values have been tabulated in various norms for laser radiation and incoherent radiation, where ocular exposure is distinguished from skin exposure. Although PAI in the eye has been demonstrated, for the purpose of this tutorial review, we consider ocular exposure to be accidental, something to be prevented by appropriate safety measures. Damage mechanisms to tissues can be classified as thermal, photochemical, and nonlinear effects, including cavitation and cell rupture.

All safety data have been compiled in tables listing the exposure limits to laser radiation, depending on wavelength, duration, and exposure area.^186^ These values have been adopted by various norms, specifically IEC60825 and ANSI Z136, of which various parts apply specifically to the use of lasers in medical settings. For low-brightness sources, such as LEDs, only thermal effects are significant, and the relevant norm is IEC62471.

**Fig. 12:**
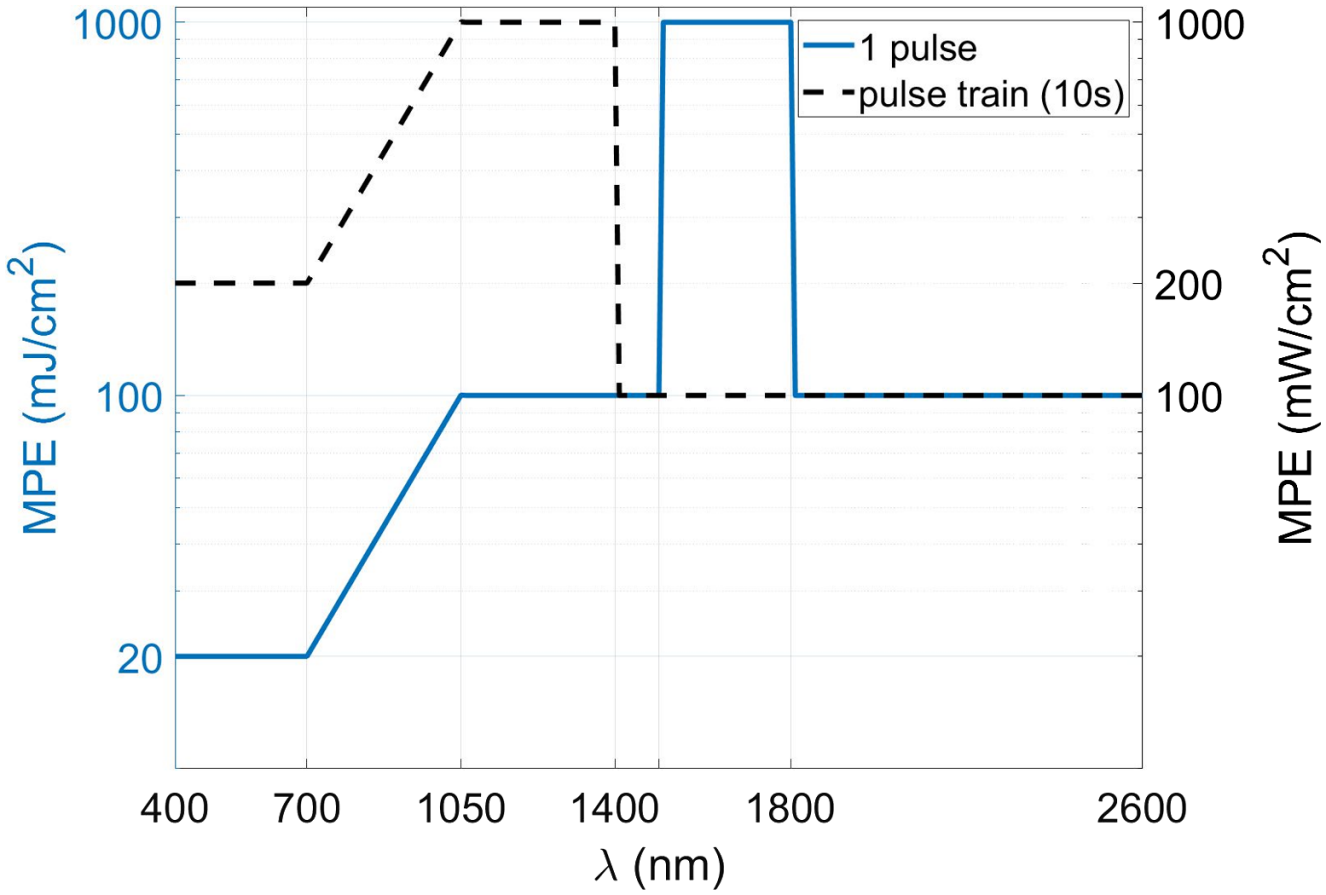
MPE as a function of wavelength for nanosecond pulses (blue line, left axis) and pulse trains (duration >10  s; black dashed line; right axis). Note the vertical scale is logarithmic.

The most important limits that are commonly applied in PAI are an MPE to nanosecond pulsed laser irradiation of $20\;\mathrm{mJ}/{\mathrm{cm}}^{2}$ for visible light (400<λ<700  nm), increasing according to $20\cdot 10^{0.002(\lambda -700)}\;\mathrm{mJ}/{\mathrm{cm}}^{2}$ in the NIR wavelength range of 700<λ<1050  nm, $100\;\mathrm{mJ}/{\mathrm{cm}}^{2}$ for wavelengths 1050<λ<1500  nm and 1800<λ<2600  nm, and $1\;J/{\mathrm{cm}}^{2}$ for 1500<λ<1800  nm. Of course, PAI is practically performed with a series of pulses, which means that the total power exposure needs to be considered as well. For pulse trains with a duration of longer than 10 s, the exposure limits are $200\;{\mathrm{mW}/\mathrm{cm}}^{2}$ (400<λ<700  nm), $200\cdot 10^{0.002(\lambda -700)}\;\mathrm{mW}/{\mathrm{cm}}^{2}$ (700<λ<1050  nm), $1\;{W/\mathrm{cm}}^{2}$ (1050<λ<1400  nm) and $100\;{\mathrm{mW}/\mathrm{cm}}^{2}$ (λ>1400  nm). The more restrictive limit of either the pulsed or CW regimes applies.^186^ The MPE for nanosecond pulses and pulse trains as a function of wavelength is shown in Fig. 12.

In the review of exposure limits, it is important to realize that these have been collected for the skin only, an organ that has evolved to be exposed to light. Endoscopic PAI or interstitial illumination will directly illuminate other tissues, and the optical exposure risk is much less studied. Systematic studies have been done for intravascular photoacoustic (IVPA) imaging only, showing evidence of photodamage only at pulse energies well exceeding the MPE for skin.^187^^,^^188^

The use of class IV laser systems (radiating their power into free space by light delivery through a handheld probe) in a healthcare setting requires strict measures for access control and mandatory eye protection for both the patient and the physician. DLs are also high-brightness sources that may require eye protection to be worn, in contrast to LEDs. These safety considerations have been obstacles to clinical use.

### 4.2 International Photoacoustics Standardisation Consortium

The availability of multiple commercial clinical systems (discussed in Sec. 3.4) necessitates international standards for ensuring the accuracy and reliability of PAI. The International Photoacoustic Standardisation Consortium (IPASC), a consortium comprised of both academic and industrial members, was established to achieve agreement on standardization with the overall goal of improving the quality of preclinical studies and accelerating clinical translation.^189^^,^^190^ To accomplish consensus in PAI standardization, four themes are defined: data management, standards development, test objects and methods, and clinical adoption.^191^

A consensus was reached on the data format to facilitate the exchange and comparison of PAI data. Where the IPASC data format has been developed to store raw time series data along with a standardized metadata structure as hierarchical data format files. For reconstructed images, commercial systems are currently working toward integration into the clinical standard digital imaging and communications in medicine format.^192^

## 5 Clinical and Translational Research

In this section, we review a concise selection of clinical and translational PAI research. We emphasize approaches that capitalize on an echo-like workflow, enabling versatility and accessibility. The examples leverage well-established acquisition technologies that are used in ultrasound echography, providing a broad perspective on the potential applications of PAI.

### 5.1 Photoacoustic Imaging for Breast Cancer

Breast PAI is an emerging modality aiming to detect and stratify breast cancer for differential diagnosis or monitoring of therapeutic effect. It exploits the elevated and inhomogeneous angiogenesis within tumoral masses and their margins.^149^^,^^193^ It has been developed as an adjuvant or stand-alone imaging modality.^194^ The currently developed breast PAI systems fall into two groups: systems using a handheld probe and dedicated breast imaging systems.

A benefit of handheld PAI systems is in their mobility and flexibility, which is a significant advantage for clinical use. In principle, such systems are not only limited to breast imaging but can also be utilized for other diagnostic purposes. The drawbacks associated with handheld devices are user dependency and limited field of view.

**Fig. 13:**
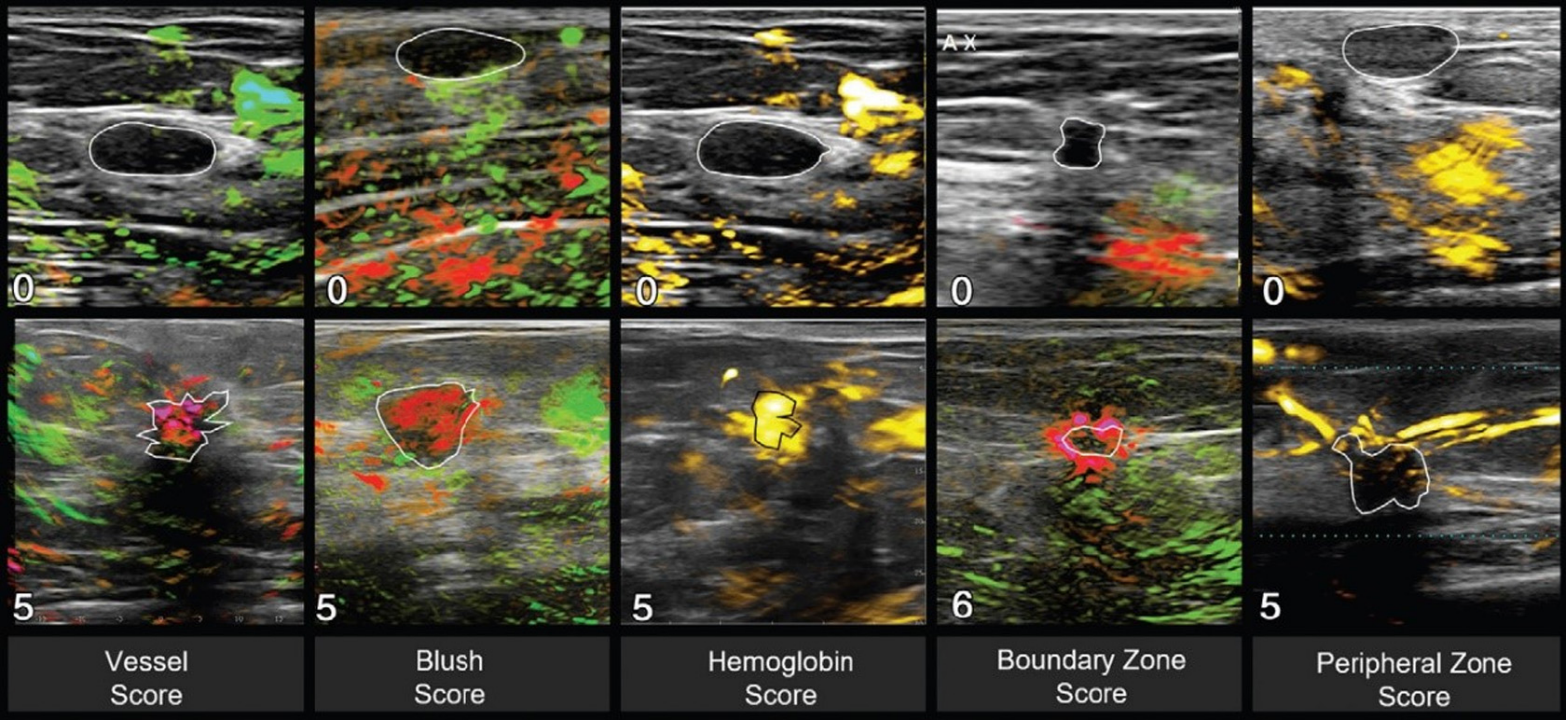
Reference images for lesions demonstrating the minimum and maximum PA feature scores. Adapted with permission from Ref. 149.

Two studies including over 2000 patients in total have demonstrated the potential for the characterization of breast tumors^149^^,^^195^ using the handheld Imagio system, introduced in Sec. 3.4. Two-wavelength PA data were acquired with a linear array to quantify tissue perfusion, blood oxygenation, and total hemoglobin. Based on these parameters, each lesion can be scored with PA features, namely vessel score, blush score, hemoglobin score, boundary zone score, and peripheral zone score, which are shown in Fig. 13.^149^ The scores were significantly higher for malignant masses than for benign and have been proven to substantially impact the radiologist’s decision in scoring the lesions.^149^^,^^195^ It was also shown that breast PA images correlate with histopathological biomarkers.^196^

Multispectral PAI at around 30 wavelengths with a curved transducer could discriminate multiple chromophores, such as ${\mathrm{HbO}}_{2}$, Hb, lipid, and water, providing additional imaging markers. For instance, the *in vivo* study demonstrated water and lipid have a fragmented appearance in cancerous regions, whereas it was homogeneous in healthy breast tissue.^193^

Dedicated breast PAI scanners aim to provide volumetric, quantitative PAI data. Recent 3D full-breast scanners provide a comprehensive, highly detailed, artifact-free overview of the vascularity, without requiring significant breast compression.^148^^,^^184^^,^^197^ Compared with handheld scanners, they are limited by their large size, cost, and complexity, requiring a dedicated space in a care facility, and significant data processing time. They also lack flexibility since they are specifically tailored for breast imaging.

### 5.2 Collagen as an Imaging Biomarker for Duchenne Muscular Dystrophy

Duchenne muscular dystrophy (DMD) is a genetic disease that causes progressive weakness and muscle degeneration, inflammation, and fibrotic transformation, eventually leading to mortality due to cardiac or respiratory failure. MRI has shown potential as a non-invasive imaging technique to quantify disease progression, however, might require sedation in uncooperative patients and is accompanied by long acquisition times. Regensburger et al.^198^ demonstrated a possible role for multispectral PAI in quantitatively detecting collagen as a biomarker for DMD, given the lack of an established objective technique to monitor upcoming treatments or pharmaceutical interventions. A prototype of the Acuity Echo (described in Sec. 3.4) was used for image acquisition in the *ex* *vivo* and *in vivo* experiments.

**Fig. 14:**
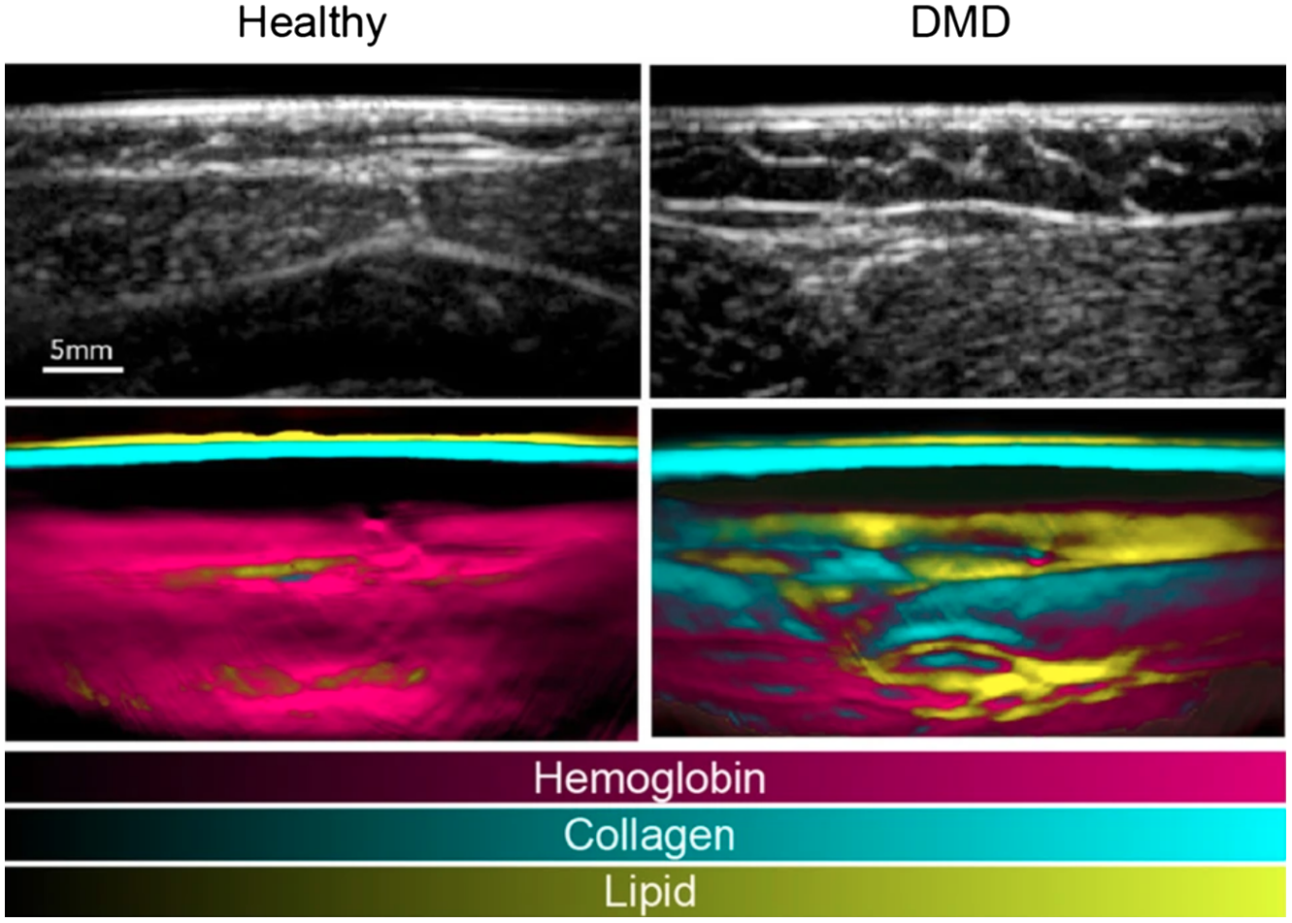
Imaging of muscle collagen as a biomarker for DMD. Top: Ultrasound echography in a healthy volunteer (left) and an individual with DMD (right). Bottom: Multispectral photoacoustic data unmixed for hemoglobin, collagen, and lipid, showing markedly higher collagen content and lipid heterogeneity as markers of DMD. Adapted from Ref. 199 and available under a CC-BY 4.0 license.

First, *ex vivo* measurement showed that the wavelength range between 660 and 1200 nm was able to differentiate collagen I, III, and IV from hemoglobin, lipid, and water. A translatable set of wavelengths 11 wavelengths was further used in a porcine study, where collagen signals could distinguish healthy from diseased muscles. Later 10 patients with DMD and 10 healthy volunteers were included in the study, where scans of eight different muscles were acquired. The spectrally unmixed collagen mean and max values in the ultrasound-based regions of interest were quantified and compared (Fig. 14). Among the different groups, all independent muscle regions showed statistically significant differences in collagen signal. Overall this exploratory study shows that multispectral PAI can be used as a contrast-free, non-invasive, fast (<7  min for eight muscle regions), and highly specific and sensitive method for DMD monitoring.

### 5.3 Intravascular Photoacoustic Imaging to Identify Vulnerable Plaque

Atherosclerosis is an inflammation-driven disease of the artery wall, in which lipids accumulate in the so-called plaques. In advanced coronary atherosclerosis, lesions may destabilize, form a thrombus, and cause acute occlusion of the vessel that results in ischemia of the heart muscle. Identification of a vulnerable plaque requires imaging of the composition, for example, large necrotic cores and thin fibrous caps likely indicate instability.^200^

A common, nonsurgical, invasive procedure to open up the occluded arteries is percutaneous coronary intervention (PCI). Intravascular ultrasound (IVUS)-guided interventions are associated with a lower risk of death, myocardial infarction, stent thrombosis, and target lesion revascularization after the placement of drug-eluting stents.^201^ In patients presenting for PCI, subsequent non-culprit lesion-related events increased with higher plaque lipid contents.^202^ This highlights the importance of intracoronary lipid imaging to detect vulnerable plaques before coronary events occur to assist risk assessment and treatment strategy. NIR spectroscopy is used for lipid core imaging, however, lacks depth information.^203^^,^^204^

**Fig. 15:**
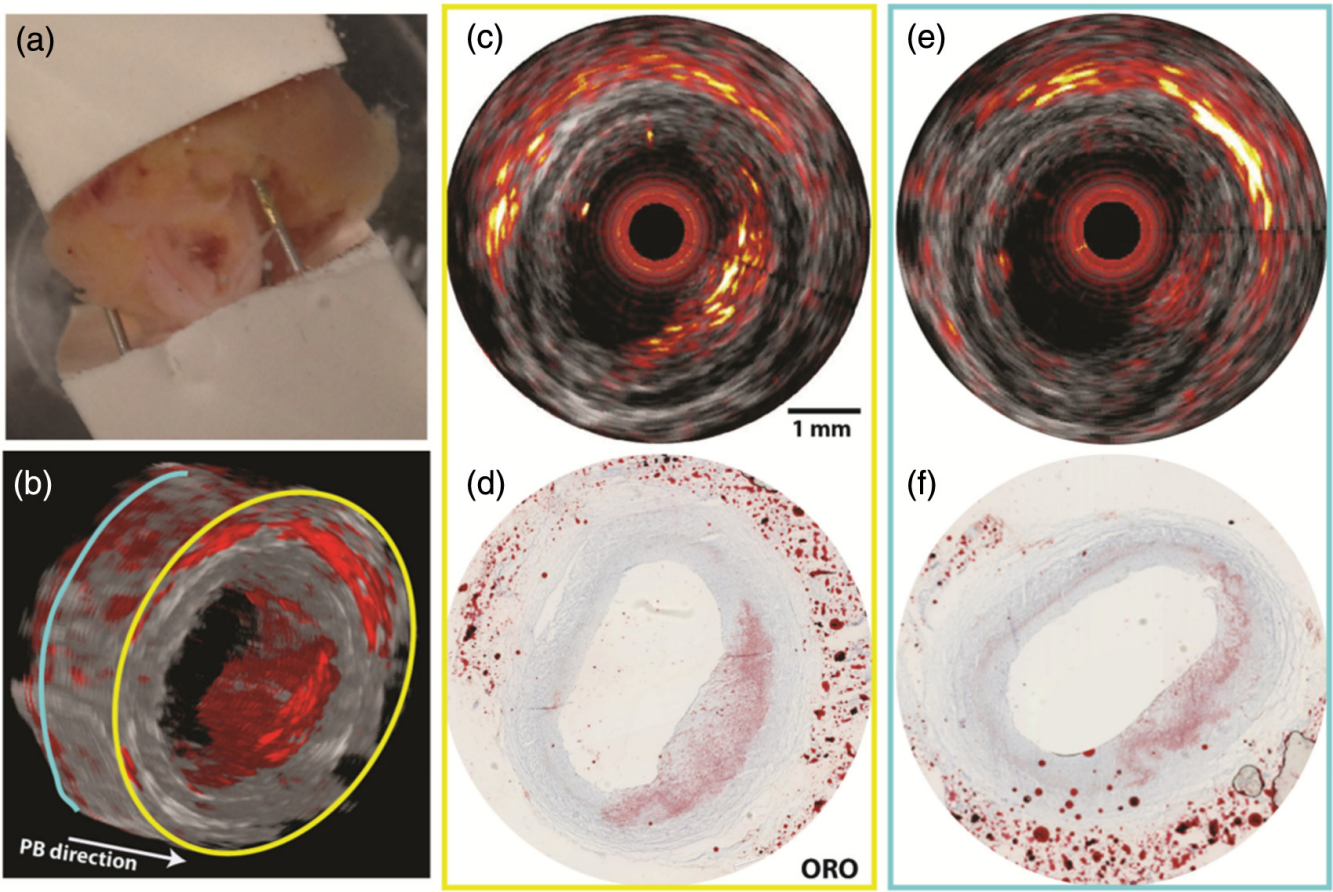
Cross-sectional IVPA images overlaid on IVUS images from a human coronary artery. (a) Imaged coronary artery fixed inside a holder. (b) 3D reconstruction of merged IVPA/IVUS pullback (PB) images. (c) and (e) IVPA images overlaid on IVUS at different locations with a dynamic range of 20 and 35 dB, respectively. (d) and (f) Histology at the corresponding image planes from (c) and (e), stained for lipids with Oil Red O (ORO). Reused with permission from Ref. 153.

IVPA imaging is an extension of IVUS, which can detect lipids, taking advantage of the distinct lipid absorption bands,^205^^,^^206^ and collocating it with structural information of the arterial wall from pulse-echo IVUS imaging.^121^^,^^207^ An IVPA/IVUS system with a 1.3 mm diameter flexible catheter is demonstrated with a sufficient clinical imaging speed of 20 frames per second.^153^ Rotational pullback recordings were obtained for an *in vivo* healthy swine model with an induced lipid target and in *ex vivo* human atherosclerotic coronary artery samples, see Fig. 15. IVPA was also applied in an atherosclerotic swine model to image coronary artery segments.^208^ The imaging of intimal lipid signals showed agreement when compared with lipid-stained histology. Further technology improvements are investigated to realize the clinical translation of IVPA in lipid-rich plaque imaging. Since 80% of the PA signal appear at frequencies below 8 MHz,^209^ a low-frequency PA acquisition would improve SNR.^22^^,^^210^

Currently, IVPA is still a preclinical technology, as the integration of adequate light delivery and sensitive PA detection in a small (1 mm outer diameter) is proving to be challenging. Many catheter designs have been published^211^^–^^216^ but to date, none of these have been clinically evaluated. The requirements on the light source (wavelength, pulse energy, and repetition frequency) have also required the engineering of custom laser systems.^19^^,^^152^^,^^217^

### 5.4 Photoacoustic-Guided Surgical Navigation

PA-guided intervention is a growing research field that is described in several reviews.^218^^–^^220^ Preclinical studies have demonstrated the feasibility of various applications, such as pedicle screw evaluation,^221^ catheter-based interventions,^222^^,^^223^ and liver surgery.^224^

**Fig. 16:**
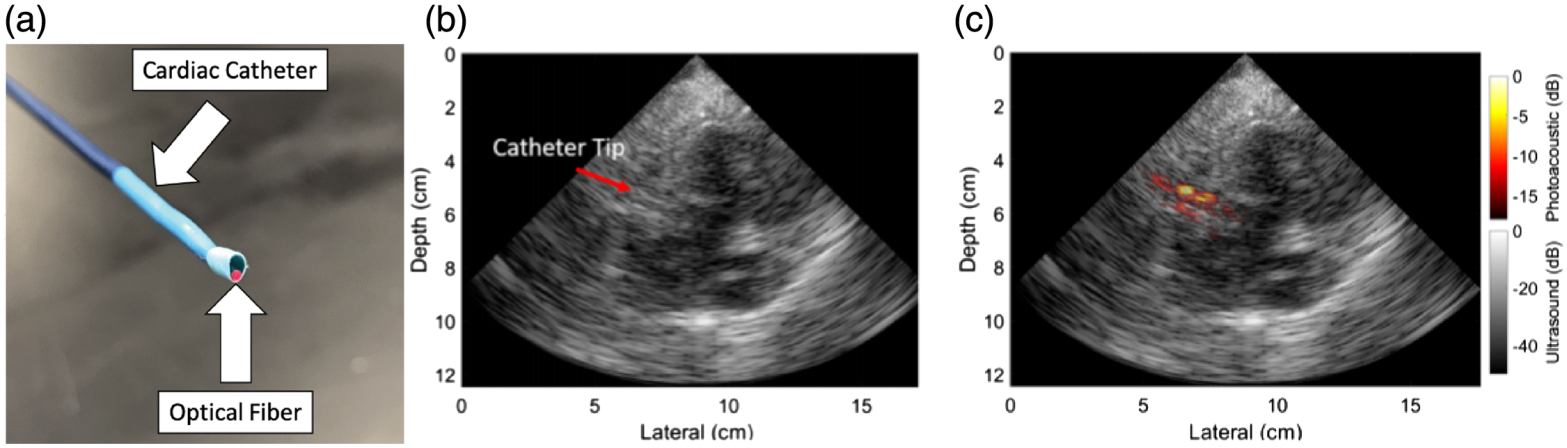
(a) A 1 mm-core-diameter optical fiber was inserted with its tip coincident with the distal tip of a 5F inner diameter, 7F outer diameter cardiac catheter. (b) Ultrasound image and (c) PA image overlaid on the corresponding ultrasound image, each showing the catheter tip in contact with the right ventricular outflow tract. The ultrasound and PA images were acquired with a subcostal acoustic window and provided depth information that is not present in the fluoroscopic images. The catheter tip location and its contact with the endocardium are more apparent in the PA image when compared to the ultrasound image. Adapted from Ref. 222, available under a CC-BY 4.0 license.

In cardiac, catheter-based interventions accurate navigation is essential for diagnosis and effective treatment. It is often performed under fluoroscopic guidance, which exposes the patient and operators to ionizing radiation. It is demonstrated that in catheter navigation, PAI could be combined with fluoroscopy to reduce exposure while adding depth information and enhancing blood vessel visualizations at depth.^222^ A research ultrasound imaging system with a linear array transducer was paired with robotic visual servoing for automatic repositioning of the ultrasound transducer. The cardiac catheter tip was localized in an *in vivo* swine model at positions within the heart and along the insertion path, see Fig. 16.

The high contrast of hemoglobin has been used to visualize major blood vessels in swine during PA-guided surgery of the liver and pancreas, which could be used to reduce intra-abdominal hemorrhage.^224^ After a laparotomy, a 5 mm fiber bundle was used to deliver light (750 nm) to the surgical workspace, whereas a robot-controlled ultrasound system was used to perform 3D imaging. Changes in the PA signal appeared when the fiber encountered large vessels; however, it is reported that out-of-plane signal and high fluence requirements remain a challenge.

### 5.5 Lymphatic Vessel Imaging for Surgical Planning

Dysfunction of the lymphatic system can lead to an accumulation of lymphatic fluid in the dermal layer of the skin, also called dermal backflow, resulting in lymphedema.^225^ Lymphovenous bypass surgery is a procedure where a lymphatic vessel located upstream of the dysfunction is anastomosed to a nearby vein, allowing lymph fluid to bypass the blockage.^226^ To locate an appropriate surgical site and determine if the patient is eligible for surgery, preoperative imaging of the lymphatic vessels and if possible nearby veins are required. A widely used method is near-infrared fluorescence lymphography (NIRF-L) using ICG as a contrast agent.^227^ However, in cases of DBF, the accumulation of ICG in the dermal layer can mask the underlying lymphatic vessels, hindering the identification of an appropriate surgical site.

PAI has been used to image these lymphatic vessels even underneath the masking dermal backflow.^11^ Where the Acuity echo system with the 3D handheld probe (discussed in Sec. 3.4) was used in a feasibility study. Images were acquired at seven wavelengths (700, 730, 760, 780, 800, 850, and 875 nm) and spectrally unmixed to distinguish hemoglobin from ICG. As a result, lymphatic and veins were identified and differentiated in real-time in 8 out of 11 patients. Next to that, in two patients, lymphatic contractility was observed. Furthermore, also the 2D probe has been used to locate lymphatic vessels and veins before lymphovenous bypass surgery.^228^

**Fig. 17:**
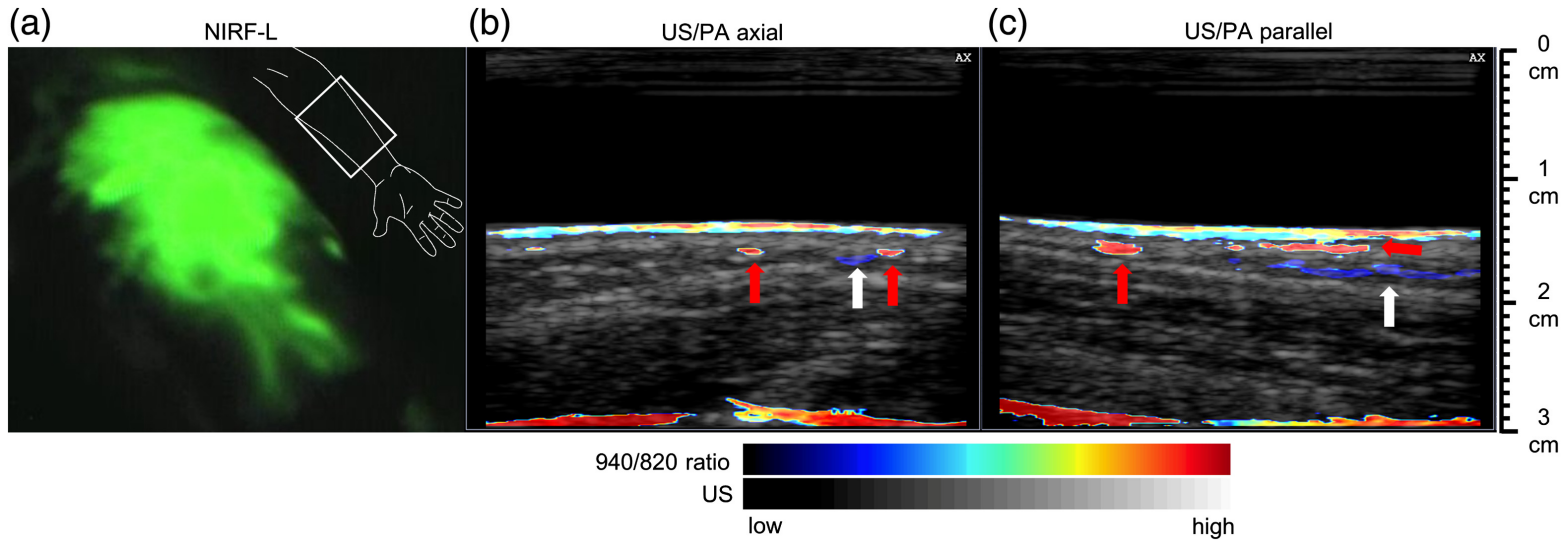
PA images of lymphatic vessels and veins overlaid on ultrasound (US) images. (a) NIRF-L image of a dorsal forearm, showing extensive dermal backflow that limits the visibility of linear lymphatic vessels. (b) and (c) Corresponding PA images overlaid on ultrasound in an axial and parallel position of the probe, red and white arrows indicate the veins and lymphatic vessels. Adapted from Ref. 168, available under a CC-BY 4.0 license.

A similar feasibility study on 15 patients was performed with the AcousticX with the 7 MHz linear array transducer, which was also discussed in Sec. 3.4, using LED illumination at 820 and 940 nm.^168^ Ratio images (940/820 nm) were used to differentiate the ICG in the lymphatic vessels from the hemoglobin in the veins. This study showed that LED-based PAI was likewise able to identify and distinguish lymphatic vessels and veins in real-time, even in the presence of DBF, as shown in Fig. 17. In the 50 imaging locations selected by NIRF-L, lymphatic vessels and veins were identified at depths up to 8.3 and 8.6 mm, with success rates of 78% and 100%, respectively.

Also, 3D, high-resolution whole-limb imaging of lymphatic vessels has been demonstrated.^229^ The clinical application in preoperative imaging is limited due to the requirement of water submersion, the complex system design, and the large size and stationary nature of the device.

## 6 Summary and Discussion

In this tutorial review, we have presented the principles of PAI from the perspective of “echo with color”: offering a unique and highly informative form of image contrast that augments the structural information offered by ultrasound echography. PAI can discriminate between many different types of tissue, perform functional imaging, and show structures that are otherwise invisible with the aid of contrast agents. All these benefits are available without the use of ionizing radiation, using an echo-like workflow. These remarkable capabilities have been developed for a number of clinical scenarios, largely in the past decade during which an imaging system integration was realized.

Relative to the volume of the scientific literature, we devoted less attention to PAM and the many deep learning studies that have been applied to PAI. PAM has found more application in pre-clinical studies than in clinical work and has been the topic of many recent reviews and tutorials.^114^^,^^115^^,^^230^ Deep learning has been applied to every step in PA image acquisition and reconstruction and has also been extensively reviewed.^99^^,^^101^

### 6.1 Challenges in Clinical Translation of PAI

PAI is at present still a research topic that is far from mainstream clinical application. We now discuss some outstanding challenges that need to be addressed before PAI can become a practical tool in the hands of clinical users. These can be broadly grouped into two interrelated categories, which we summarize as (1) image quality and interpretation and (2) ease of use and accessibility.

#### 6.1.1 Image quality and interpretation

With most of the currently available technology, the interpretation of PA images is less straightforward than that of, for instance, echography. Fundamentally, the reasons for this difference originate in the limitations of PA image acquisition and reconstruction. We outline three properties of PA that complicate the understanding of images.

First, the target of interest may be relatively weak compared with features that are not of interest. Unlike echography, PA signals tend to have a large dynamic range. Near the surface, or wherever the light is administered, a minute amount of absorption may create very strong signals because of the large fluence. Optical attenuation can reduce the energy available for PA excitation by three orders of magnitude or more in a few centimeters of imaging depth, rendering the signal generated by strong absorbers relatively weak. Ultrasound receiving instrumentation must match that dynamic range, being able to detect weak signals and posing stringent requirements on the AFE of the ultrasound system, which in clinical ultrasound systems tend to be highly optimized for echo imaging,^147^^,^^231^ with a limited number of parallel acquisition channels and limited control over the front end gain.

Second, we discussed the many forms of image artifacts that occur in PAI in Sec. 2.7, creating ghost features that need to be recognized when interpreting images. These derive from the large dynamic range discussed above (out-of-plane clutter), tissue optics (fluence coloring), acoustic heterogeneity (aberration), and acoustic detection (unwanted echoes and band limitation). Most importantly, finite transducer aperture, element size, and directionality cause prominent limited-view artifacts (comet tails and side lobes). Compared with ultrasound echography, where images are formed from reflections of a directional beam, PA waves are much more omnidirectional. The geometry offered by linear array or phased array transducers, the most common types of handheld ultrasound probes, is highly suitable for pulse-echo imaging but not optimal for receiving PA signals. Many PAI experiments aim to visualize blood vessels, contrast agents in tumors, needles, or catheters finding a target, or similar sparse structures. It can be argued that the imaging and reconstruction artifacts are more visible than in, for instance, echo images that exhibit a speckle background in most tissues, masking artifacts.

Third, beyond interpretation, these artifacts affect image quantification. The retrieval of absolute absorption coefficients is one of the major goals of PAI. It is important to realize that the accuracy with which absolute quantification can be achieved is strongly dependent on the characteristics of the acquisition hardware, particularly transducer geometry and receive bandwidth, on the image reconstruction algorithm that is chosen, and on the knowledge of optical and acoustic parameters that can be incorporated in such a reconstruction. In the presence of spectral coloring, even relative quantification based on ratiometric spectroscopy or spectral unmixing can be impaired, and detailed modeling of tissue optics becomes essential.

PAI systems for imaging specific anatomies, most prominently the breast,^148^^,^^197^^,^^232^ lower extremities,^229^ and brain,^18^ have been realized. They create highly detailed maps of vascular structures by acquiring signals at a large solid angle, using highly sophisticated image reconstruction methods. This approach mitigates many of the concerns outlined above but sacrifices broad applicability and flexibility. A middle ground is struck by the use of concave handheld arrays, either in an arc-^13^^,^^67^^,^^198^ or cup-shaped transducer.^66^ These systems allow for a more faithful reconstruction of the source pressure than linear arrays do, although their imaging characteristics degrade away from the focal zone^233^ and the probes are more bulky than standard echo probes.

#### 6.1.2 Ease of use and accessibility

The ideal clinical PAI system is just as easy to use as an echo machine: mobile, with a probe that is easy to handle, a real-time, video-rate image display, and controls that allow the user to straightforwardly dial in the optimal settings for an easily interpretable image. PAI is not at that point yet. Many PAI systems require the use of laser goggles and a dedicated room with laser warning signs and access control. Such infrastructure does not exist in most healthcare facilities. Active cooling of the laser can cause the imaging system to be noisy. PAI probes are often large and cumbersome to manipulate because of stiff fiber bundles included in, or attached to, the transducer cable. Instruments that integrate the light source in the probe circumvent many of these issues, but generally deliver reduced imaging depth and less wavelength flexibility due to lower pulse energy and longer pulse duration.^80^^,^^160^ This may limit their application space. All these factors affect usability and thus clinical uptake.

Impact starts with accessibility. A high-power laser can be a very expensive subsystem to include in an imaging device, especially if tunability is required. High costs can substantially slow the spread of a technology beyond a research setting. The availability of expensive clinical technologies is often limited to a few high-tech centers, which specialize in advanced care for more complex diseases or patients, and thus smaller groups.

In a research setting, the objective is often to demonstrate clinical benefit of a new technology compared with the standard of care. Benefit is only part of the equation, however. Health technology assessment (HTA) is a family of methods to determine the actual value of an innovation in clinical care, a form of cost-benefit analysis that quantifies the gain in health attributable to the new technology, compared with its cost.^234^ So-called “early HTA” applies HTA methods to technologies that are still in the preclinical or early clinical testing phase, using scenarios for costs and benefits.^235^^,^^236^ HTA is a critical step for adoption and reimbursement and may contribute to a sustainable healthcare system.^237^

In the case of PAI, it is important to realize that health benefits are not achieved by diagnostic technology alone.^238^ A therapeutic intervention, using the PA information, is required to achieve the actual benefit. The additional cost needs to be compared with the essential benefit realized by the application of PAI in the clinical decision-making process must be determined, which likely requires outcomes studies using hard endpoints (event-free survival, for instance). Such studies have not been performed. As far as we are aware, no (early) HTA studies of PAI have been published to date. Assuming that cost increases with system complexity, it is likely that relatively simple systems that can be deployed in different diagnostic scenarios will need to meet a lower bar for the health benefits they realize, to be considered cost effective.

### 6.2 Outlook

Scientists in PA research have unlocked a wealth of biological phenomena using the technique. The past decade has been incredibly exciting, with the emergence of integrated PA/ultrasound,^147^ advanced chemistry producing targeted contrast agents,^173^^,^^239^ super-resolution imaging,^240^^–^^242^ and the introduction of clinically approved imaging systems (see Sec. 3.4), to name just a few developments. The publication of dozens of studies involving patients, some seriously challenging established knowledge that is based on older imaging techniques,^198^ signifies that PAI is headed for the clinic. These developments will undoubtedly continue: PAI is a vibrant, fast-moving field with a mind for innovation, aiming to create new forms of diagnostic imaging and enable new therapies.

To actually deliver on that ambition, the challenges that we discussed above will have to be addressed. Actual, real-world impact requires that PAI be simple to use and interpret, be robust, fast, safe, and cost effective (preferably even cheap!). An impetus in translation-oriented technical research and, particularly, engineering is needed to realize this. Highly sensitive, multichannel ultrasound detectors with a large opening angle would detect the weakest signals. Robust light sources that deliver light at biologically relevant wavelengths will need to be invented, sacrificing tunability for efficiency, speed, and affordability. Ideally, PAI works with light sources that do not require laser safety precautions. Real-time image reconstruction algorithms, creating high-contrast, readily interpretable images, will need to run fast on conventional computing hardware. If the PA community dedicates part of its resources and ingenuity to combining existing but previously untested technologies, and researching new ones, these challenges can be met. This effort needs to pay attention to practical utility at every step.

Because of its versatility, many clinical needs can potentially be addressed using PAI. We have highlighted a few areas in Sec. 5 in a list that aims to diversify rather than exclude. PAI has the potential to quantify tissue chromophore concentrations, but this is not generally possible in most limited-view geometries that are amenable to routine clinical deployment. Promising applications of PAI apply qualitative images that can be straightforwardly interpreted to locate otherwise hidden features, such as blood and lymph vessels, plaque lipids, or surgical instruments. Such point-like or linear structures can be reconstructed with imaging algorithms methods that capitalize on sparsity and can be visualized with a small dynamic range, suppressing imaging artifacts. Alternatively, image classifiers may be developed to support the interpretation of multiparametric data (such as those in Fig. 13), based on physical models or heuristics. Finally, spectroscopic PAI of tissue volumes, with adequate modeling of tissue optics to account for spectral coloring, can be an extremely powerful tool for tissue characterization. The upshot of all of these computationally empowered PAI approaches is that the interpretation of images is simplified and in the end should not require intimate knowledge of the underlying physics.
